# Communicating the health implications of global environmental change: a mixed-methods systematic review of health framing in environmental messaging

**DOI:** 10.1093/abm/kaag002

**Published:** 2026-02-12

**Authors:** Frederick H F Chan, Rachel W S Koh, Steve H L Yim, Benjamin P Horton, Konstadina Griva

**Affiliations:** Department of Social Work and Social Administration, The University of Hong Kong, Hong Kong, 000000, China; Lee Kong Chian School of Medicine, Nanyang Technological University, 308232, Singapore; Lee Kong Chian School of Medicine, Nanyang Technological University, 308232, Singapore; Centre for Climate Change and Environmental Health (CCEH), Nanyang Technological University, 639798, Singapore; Asian School of the Environment, Nanyang Technological University, 639798, Singapore; Earth Observatory of Singapore, Nanyang Technological University, 639798, Singapore; School of Energy and Environment, City University of Hong Kong, Hong Kong, 000000, China; Lee Kong Chian School of Medicine, Nanyang Technological University, 308232, Singapore

**Keywords:** framing, climate change, health communication, systematic review

## Abstract

**Background:**

Global environmental change poses a significant threat to human health, necessitating effective communication strategies to raise public awareness and motivate mitigation and adaptation actions. Previous studies have examined whether framing climate change and other environmental issues as health problems can increase public engagement, with mixed results.

**Purpose:**

This mixed-methods systematic review synthesizes existing evidence on the effectiveness of health framing in text-based environmental communication interventions.

**Methods:**

We searched 5 electronic databases (Web of Science, Scopus, PubMed, PsycINFO, and Communication and Mass Media Complete) from inception to May 9, 2025, and identified 46 relevant articles (54 studies). The Mixed Methods Appraisal Tool was used to assess study quality, and qualitative narrative synthesis was performed.

**Results:**

Most studies were randomized controlled trials conducted among the general public in high-income, English-speaking countries. Key findings indicate that health-framed environmental messages are generally perceived as clear and helpful, particularly when employing a gain frame and emphasizing mitigation benefits. Health framing also effectively increases threat perception, policy support, and health-protective intentions, although its impacts on sustainable lifestyle changes and advocacy behaviors are less consistent.

**Conclusions:**

Future research should incorporate rigorous designs and diverse populations and focus on long-term, real-world outcomes to obtain a clearer understanding of effective communication strategies at the intersection of global environmental change and health.

**Registration:**

The systematic review protocol was registered within the PROSPERO database (CRD420251050978).

## Introduction

Multiple global environmental changes (GECs), including climate change, pollution, biodiversity loss, soil degradation, and ocean acidification, are underway, with substantial and complex implications for human health.^1^ Climate change, the best-known among these trends, is increasingly recognized as a threat to global health,^1^^,^^2^ with evidence suggesting that climate mitigation actions can yield public health benefits.^3^ Despite this scientific consensus, research on public awareness and understanding of these issues remains limited. Existing evidence, predominantly from high-income, English-speaking countries, indicates rising public recognition of climate change as a health threat, though very few can list specific health impacts.^4^ Health professionals also view climate change as a serious health concern but often lack in-depth knowledge.^4^

Effective communication about the intersection of GECs and health is crucial to inform and prepare the public to adapt or respond to these challenges.^5^ The metatheory of health communication offers an integrative framework for how messages shape behavior through multiple pathways.^6^ According to the model, health communication first influences a range of antecedents including knowledge, skills, cognitive appraisals (eg, attitudes, perceived threat), emotional reactions (eg, fear), and social-contextual factors (eg, collective efficacy), which in turn drive behavioral intentions and, ultimately, actions that contribute to health outcomes.^6^ When applied to GECs, health-framed messages can raise awareness of environmental issues, increase threat perception, elicit emotions such as worry and fear, and shift attitudes toward climate solutions, thereby influencing the likelihood that individuals: (1) adopt personal protective behaviors (eg, mask use during air pollution events), (2) embrace sustainable lifestyle choices that yield health and environment co-benefits (eg, plant-based diet), and (3) engage in climate advocacy and policy support that accelerate systemic change. Health framing is particularly well suited to the communication of GECs because it connects planetary changes to the universally valued domain of human health, translating abstract climate trends into concrete, proximate health threats.^7^^,^^8^ In contrast, traditional environment-focused narratives, which center on the planetary implications of GECs, are often complex and lack personal relevance, thereby limiting engagement across diverse audience groups.^9^

However, previous research on health framing in environmental communication has yielded mixed results. For instance, while Dasandi et al. found that highlighting the health implications of climate change and mitigation strategies boosted participants’ support for climate policies,^9^ Bernauer and McGrath observed no significant impact of health framing over climate risk framing on policy support or advocacy intentions.^10^ Evidence in this field is burgeoning yet fragmented, with research adopting a variety of study designs, message interventions, and outcome measures. This diversity, while enriching the field, poses a challenge in drawing conclusive insights and formulating definitive recommendations for effective communication strategies that address the intersection of GECs and health. While a literature review has been published,^5^ the authors acknowledged that a comprehensive systematic search was not performed.

To address this gap, this mixed-methods systematic review aims to synthesize research on the effectiveness of health framing in text-based environmental communication. While previous research has primarily focused on health framing in climate communication,^5^ in this review, we adopt a broader scope by examining health framing in messages about any aspect of GECs, such as climate change, biodiversity loss, and renewable energy policies. Additionally, while some studies have examined health framing in complex communication interventions such as videos and maps,^11^ our review focuses exclusively on plain text message interventions, which would allow for a clearer understanding of the isolated effects of written information, minimizing confounding effects from other communication mediums such as images and animations. As such, this review examines (1) individuals’ reactions to health-framed environmental messages, (2) differences between health frame and other frames in influencing emotional, cognitive, and behavioral outcomes, and (3) potential moderators and mediators of the health framing effects. We will also outline research gaps and offer recommendations for future research and future communication strategies.

## Methods

The systematic review protocol was registered within the PROSPERO database (CRD420251050978). Results were reported following the Preferred Reporting Items for Systematic reviews and Meta-Analyses (PRISMA) guideline.^12^^,^^13^

### Eligibility criteria

Studies were included if they (1) presented participants with plain text messages focusing on environmental issues or policies (eg, climate change, net zero policy), (2) used a health frame (ie, emphasizing the health implications of environmental issues or policies) in at least one group of participants, and (3) evaluated participants’ responses to these messages. In this review, we defined framing as “an emphasis on different aspects of an issue with the goal of shaping people’s opinion about the issue.”^7^

Studies were included regardless of language, design, or population. However, studies were excluded if they used non-text communication mediums like figures and videos. Studies were also excluded if messages were not focused on an environmental issue, but instead focused on the health and environmental implications of lifestyle behaviors (eg, plant-based diet) or food products (eg, red meat). In addition, studies where health outcomes of environmental issues were mentioned, but not as the primary focus of the message (eg, health effects mentioned alongside other outcomes), were excluded. Unpublished studies and gray literature were excluded.

### Information sources, search strategy, and selection process

The following databases were searched from inception to August 16, 2024: Web of Science Core Collection, Scopus, PubMed, PsycINFO, and Communication and Mass Media Complete. The search terms included exact words or synonyms of: framing, text message, essay, health, co-benefit, global environmental change, climate change, and global warming. Subject headings such as “framing effects” and “climate change” were also used. A sample search strategy is provided in Supplementary Material S1.

Two authors (F.C. and R.K.) independently screened titles and abstracts in Covidence (https://www.covidence.org) to exclude irrelevant studies. Full texts of the remaining articles were also independently assessed to determine eligibility, with disagreements resolved by a third reviewer (K.G.). Interrater reliability was excellent (kappa = 0.982). To ensure comprehensiveness and recency of the review, reference lists of included articles were scanned for relevant studies, and an updated search was conducted to retrieve records published between the initial search date and May 9, 2025.

### Data extraction

The following data were extracted from included studies to a Microsoft Excel spreadsheet: author; year of publication; study objective; study location; study design and data collection methods; participant characteristics; characteristics of message interventions; comparators (if applicable); and main findings. Data extraction was performed independently by F.C. and R.K., with disagreements resolved by discussion with K.G. Authors were contacted if there was incomplete information.

### Quality assessment

We assessed the methodological quality of included studies using the Mixed Methods Appraisal Tool (MMAT),^14^ which provides study design-specific items to evaluate quantitative randomized controlled trials (RCTs), quantitative non-randomized studies, quantitative descriptive studies, qualitative studies, and mixed-methods studies. F.C. and R.K. independently appraised each study, with discrepancies resolved through discussion.

### Data synthesis

The characteristics of studies and message interventions were summarized descriptively. Qualitative narrative synthesis was performed, given the mixed study designs included in this review. Specifically, we synthesized findings on the effectiveness of health framing across 5 groups of outcomes: message evaluations (eg, comprehensibility of messages), emotional reactions (eg, worry), cognitive responses (eg, perceived threat), policy attitudes (eg, policy support), and behavioral outcomes (eg, advocacy intentions). Potential moderating and mediating effects were also explored. However, statistical synthesis was not possible due to the heterogeneity in study designs, interventions, outcomes, and analysis methods.

## Results

### Study selection

**Figure 1:**
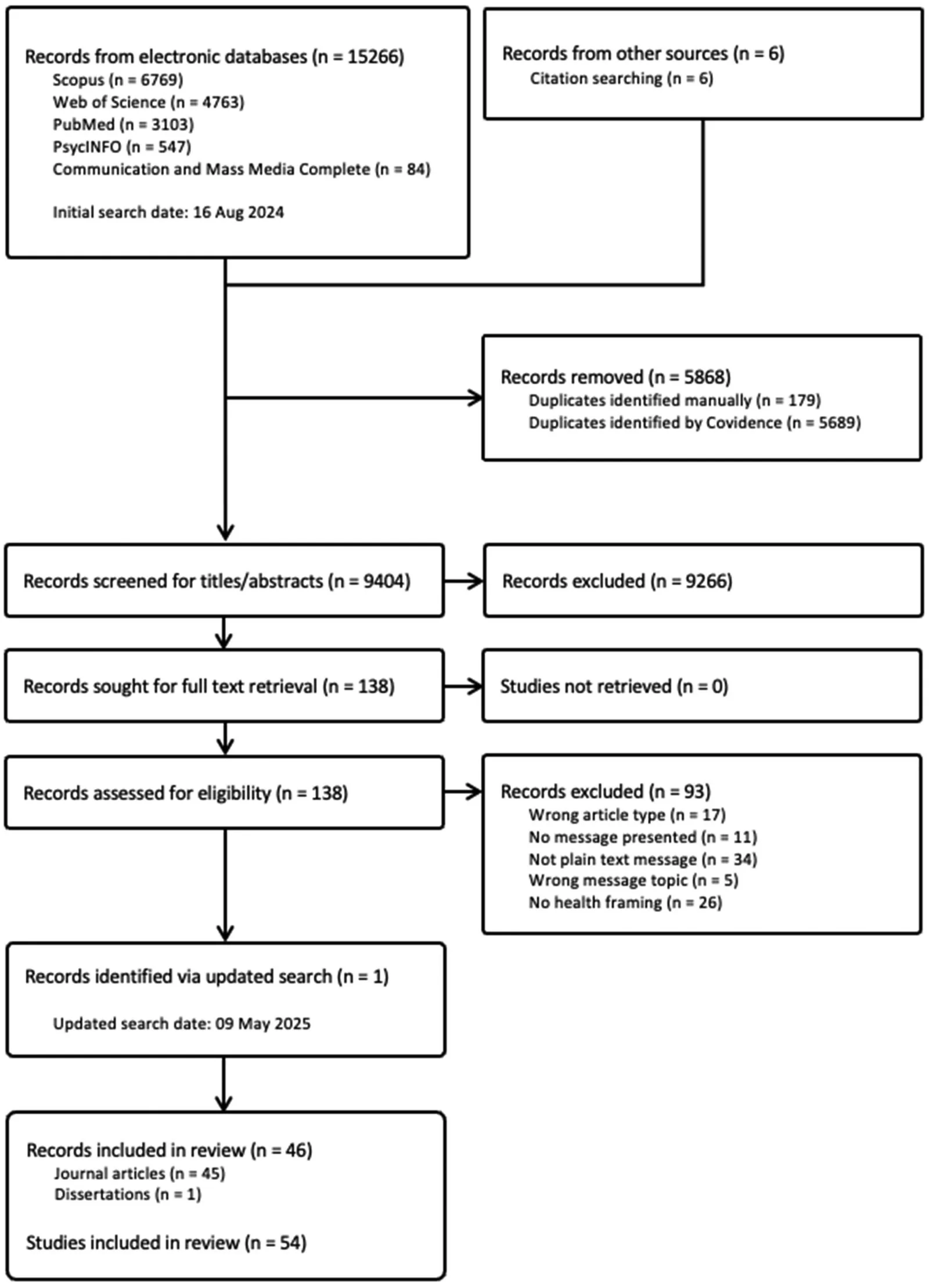
PRISMA flowchart.

The initial search (August 16, 2024) retrieved 15 266 records from electronic databases, and 6 records were identified through citation searching. After screening the titles and abstracts and removing duplicates, 138 papers were retrieved and assessed for eligibility, of which 45 met the inclusion criteria. An updated search (May 9, 2025) identified 1 additional relevant article. Thus, a total of 46 papers (54 studies) were included in this review. The screening process according to the PRISMA guideline is summarized in Figure 1.^13^

### Study characteristics

Table 1 summarizes the characteristics of included studies. More detailed information on each study and the full reference list of included studies are presented in Supplementary Material S2. Although our search included articles in languages other than English, eventually all included studies were in English. The included papers were published from 2010 onward, with a notable increase since 2015. Most studies were conducted in high-income, English-speaking countries, particularly the United States (*n* = 37, 68.5%), the United Kingdom (*n* = 3, 5.6%), and Canada (*n* = 2, 3.7%). The 54 studies consisted of 44 quantitative RCTs (81.5%), 6 quantitative non-randomized studies (11.1%), 2 mixed-methods studies (3.7%), and 2 qualitative studies (3.7%). Sample sizes ranged from 16 to 102 556 participants, totaling 183 486 across all studies. Most studies focused on the general public (*n* = 40, 74.1%) and student populations (*n* = 7, 13.0%).

**Table 1:** Summary of study characteristics.

Domain	Study characteristics	No. (%) of studies (*N* = 54)
**Study design**	Quantitative randomized controlled trial	44 (81.5)
	Quantitative non-randomized study	6 (11.1)
	Mixed-methods study	2 (3.7)
	Qualitative study	2 (3.7)
**Study location**	United States	37 (68.5)
	International	4 (7.4)
	Mainland China	3 (5.6)
	United Kingdom	3 (5.6)
	Canada	2 (3.7)
	France	1 (1.9)
	Iran	1 (1.9)
	Poland	1 (1.9)
	South Korea	1 (1.9)
	Taiwan	1 (1.9)
**Year**	2010-2014	6 (11.1)
	2015-2019	22 (40.7)
	2020 onwards	26 (48.1)
**Target population**	General public	40 (74.1)
	Students	7 (13.0)
	Health professionals	4 (7.4)
	Netizens	2 (3.7)
	Pregnant women or mothers of young children	2 (3.7)
	Childcare providers	1 (1.9)
	Older adults	1 (1.9)
	People with low income	1 (1.9)
	Politicians	1 (1.9)
**Sample size**	0-100	5 (9.3)
	101-1000	33 (61.1)
	>1000	16 (29.6)

Not all authors provided the full stimuli used in their studies. Based on the available information, the health messages used varied in length from 14 to 1200 words, with most under 500 words. The majority of studies focused on climate change (*n* = 30, 55.6%), presenting participants with messages that described the health consequences of climate change (*n* = 6),^11^^,^^15–19^ the health benefits of climate solutions (*n* = 9),^10^^,^^20–26^ or both (*n* = 15).^9^^,^^27–37^ Some other studies used messages that focused on pollution (*n* = 21, 38.9%).^20^^,^^38–54^ A smaller portion of studies presented messages about the health impacts of other environmental issues, such as biodiversity loss, ocean acidification, and marine ecosystem change.^55–57^

Health-framed environmental messages were frequently compared with other frames, including the environment frame (ie, emphasizing implications for nature and ecosystems),^9^^,^^10^^,^^18^^,^^19^^,^^21^^,^^22^^,^^24–26^^,^^28^^,^^30^^,^^36^^,^^39^^,^^42^^,^^43^^,^^46^^,^^47^^,^^53^ economic frame (ie, emphasizing economic implications in terms of jobs, investments, markets, and industry),^9^^,^^21^^,^^25^^,^^30^^,^^41^^,^^47^^,^^53^ national security frame (ie, focusing on threats to human security as a result of violent conflict and human migration),^30^^,^^35^^,^^36^ conflict frame (ie, emphasizing political disputes over climate change),^30^ moral frame (ie, highlighting ethical calls to action),^30^ and social norm frame (ie, focusing on scientific consensus and public belief in climate change).^20^^,^^27^^,^^31^ Some studies also examined alternative health framing approaches, such as comparing gain versus loss framing,^21^^,^^37^^,^^51^ high- versus low-risk framing,^37^^,^^50^^,^^55^ or high versus low personal relevance framing.^52^

### Quality assessment

The quality ratings of each study are presented in Supplementary Material S3. Among the 44 quantitative RCTs, only 8 (18.2%) met 3 or more of the 5 methodological quality criteria, with none fulfilling all criteria. Most RCTs lacked detail on randomization, baseline comparability between groups, and blinding of outcome assessors. Although many RCTs in this review were embedded in online surveys, which typically ensure proper randomization and blinding through automatic algorithms, this information was rarely reported explicitly. Additionally, around 45% of RCTs did not provide information on manipulation or attention checks, making it difficult to confirm participants’ adherence to the assigned interventions. In contrast, the quality of other study types was notably high. All quantitative non-randomized studies (*n* = 6), mixed-methods studies (*n* = 2), and qualitative studies (*n* = 2) met al.criteria relevant to their respective study designs.

### Results of synthesis

This section reports synthesized findings on the effectiveness of health framing across 5 groups of outcomes: message evaluations, emotional reactions, cognitive responses, policy attitudes, and behavioral outcomes. Table 2 provides a summary of the synthesized findings, indicating the direction of health framing effects observed within and between groups for each outcome.

**Table 2:** Summary of synthesized findings regarding the health framing effects.

Outcome		Pre-post change after reading	Compared to no-message control	Compared to other active frames	Compared to alternative health frames
**Message evaluations**					
	Positive evaluations (clear and helpful)	Increased from the opening to the concluding section of the message (1 study)	N/A	Insufficient data	Increased when using gain framing compared to loss framing (4 studies)
**Emotional reactions**					
	Hope	Insufficient data	No difference (1 study)	No difference (1 study) or decreased (1 study) when compared to environment frame; No difference when compared to national security frame (2 studies)	Increased when using a threat-efficacy structure (1 study)
**Cognitive responses**					
	Perceived threat	Increased (3 studies)	Increased (1 study)	Increased when compared to environment frame (2 studies); Increased when compared to economic frame (1 study)	Increased when emphasizing personal relevance (1 study); Decreased when message is catastrophic (1 study)
	Perceived efficacy	Increased (1 study)	Increased (1 study) or no difference (1 study)	No difference when compared to national security frame (1 study)	Insufficient data
**Policy attitudes**					
	Support for environmental policies and government action	Increased (1 study)	Increased (4 studies) or no difference (1 study)	Increased (6 studies) or no difference (4 studies) when compared to environment frame; Increased when compared to economic frame (4 studies)	Increased when mentioning opponents to climate action (1 study)
**Behavioral outcomes**					
	Sustainable lifestyle behavior (intention)	Insufficient data	No difference (1 study)	No difference when compared to environment frame (4 studies); No difference when compared to national security frame (1 study)	Insufficient data
	Sustainable lifestyle behavior (actual)	Insufficient data	Insufficient data	Insufficient data	Insufficient data
	Climate advocacy behavior (intention)	Increased (3 studies)	No difference (1 study)	No difference when compared to environment frame (4 studies); No difference when compared to national security frame (1 study)	Increased when emphasizing health benefits of climate solutions (1 study); Increased when mentioning opponents to climate action (1 study)
	Climate advocacy behavior (actual)	Insufficient data	No difference (2 studies)	No difference (4 studies) or decreased (2 studies) when compared to environment frame	No difference when including normative information (2 studies); No difference when mentioning opponents to climate action (1 study); No difference when using a gain frame (1 study)
	Health-protective behavior (intention)	Insufficient data	Increased (1 study)	Increased when compared to environment frame (1 study)	Increased when emphasizing the easy precautions one could take to protect themselves (1 study); Increased when emphasizing public health risks compared to personal health risks (1 study)
	Health-protective behavior (actual)	Insufficient data	Increased (2 studies)	Insufficient data	Insufficient data

#### Message evaluations

Ten studies assessed participants’ evaluations of health-framed environment messages.^11^^,^^16^^,^^23^^,^^30^^,^^34^^,^^37^^,^^38^^,^^42^^,^^49^ These included quantitative studies where participants rated aspects of the messages such as comprehensibility, relevance, and level of agreement,^16^^,^^37^^,^^38^^,^^42^^,^^49^ as well as qualitative studies where participants provided feedback and recommendations after reading the messages.^11^^,^^23^^,^^34^ Additionally, 2 studies measured the time spent by participants reading health-framed climate messages as an indicator of their interest.^30^

Four quantitative studies compared message evaluation outcomes between different frames.^30^^,^^37^^,^^42^ One study found that health-framed climate articles garnered longer reading times (ie, greater interest) than economic, morality, and national security frames, though not when compared with environment or conflict frames.^30^ This effect was also diminished when climate articles were presented amidst other non-climate–related articles.^30^ Another RCT presented messages about the impacts of power plant emissions and showed no difference in participants’ agreement levels between health and environment frames.^42^ In addition, 1 study indicated that a message about climate mitigation and health effects was evaluated more negatively (ie, boring, exaggerated, hard to understand) when using a loss frame than when using a gain frame.^37^ Other quantitative studies compared message evaluation outcomes across specific categories of health-framed messages.^16^^,^^38^^,^^49^ For instance, information on mental health impacts of global warming was perceived as more novel but less comprehensible and relevant than information on physical health effects such as those due to exposures to extreme heat and air pollution.^16^

Interestingly, a mixed-methods study observed an increase in participants’ positive evaluations (ie, clear and helpful) from the opening to the concluding section of a health-framed climate essay, suggesting that the effect of health framing may become more pronounced after people have time to consider the evidence.^34^ Also, sentences describing health benefits of mitigation strategies in this essay were seen as clearer and more useful than those describing health risks of climate change.^34^ In line with these findings, 2 qualitative studies highlighted that messages emphasizing the health benefits of climate action were seen as particularly helpful.^11^^,^^23^ Participants in these 2 studies also suggested that climate health messages should include credible statistics, stories and lived experiences, and actionable recommendations at both individual and systemic levels.^11^^,^^23^

In summary, while evidence on differential evaluations of health-framed environmental messages versus other frames is limited, health-framed messages are generally perceived as clear and helpful, especially when using a gain frame and highlighting mitigation benefits.

#### Emotional reactions

Eight RCTs assessed emotional reactions to health-framed environmental messages.^16^^,^^28^^,^^32^^,^^35–37^^,^^52^^,^^55^ The most frequently assessed emotion was hope, with mixed findings across 4 studies. Two studies in the general population found no effect of health framing on hopeful emotions compared to environment framing, national security framing, or a no-message control condition.^35^^,^^36^ Conversely, among high school agriculture students, a health frame resulted in less hope compared to agriculture and environment frames.^28^ In university students, a health-framed message about climate mitigation evoked more hope than fear, particularly when using a gain frame and when the message was preceded by a threat-based message highlighting climate health risks.^37^

Overall, health-framed climate messages following a threat-efficacy structure may elicit hopeful emotions, though evidence for superiority over other frames is limited. Due to limited data and heterogeneity in study populations and message designs, findings on other emotions such as worry (2 studies), anger (2 studies), and fear (2 studies) cannot be synthesized.

#### Cognitive responses

Cognitive responses were assessed in 15 studies,^16^^,^^32^^,^^33^^,^^35^^,^^39–41^^,^^46^^,^^48^^,^^49^^,^^52^^,^^55–57^ covering constructs such as perceived threat (8 studies),^16^^,^^33^^,^^41^^,^^46^^,^^48^^,^^49^^,^^52^^,^^55^ perceived efficacy (2 studies),^35^^,^^48^ and perceived importance of climate change (2 studies).^16^^,^^35^

Perceived threat was operationalized in various ways, including perceived susceptibility, perceived severity, perceived harm to self, and perceived harm to future generations. Three studies found a significant increase in participants’ threat perception after exposure to health-framed environmental messages.^16^^,^^48^^,^^49^ Four RCTs consistently showed higher perceived threat in participants exposed to health frames compared to environment frame,^33^^,^^46^ economic frame,^41^ and no-message controls.^41^^,^^48^ Emphasizing personal relevance in health frames (ie, by framing the risk as more geographically proximate) also increased threat perception.^52^ However, in 1 study framing biodiversity loss as a health issue, threat perception was attenuated, unexpectedly, in the high-risk condition (ie, emphasizing the risk of biodiversity loss to health) compared to the low-risk condition (ie, same factual information but less catastrophic).^55^

Regarding perceived efficacy, one RCT found that pregnant women’s response efficacy and self-efficacy increased after exposure to daily messages about the health risks of air pollution and protective behaviors, compared to the no-message control group.^48^ In contrast, another study in the general population found no difference in perceived efficacy across groups exposed to health frame, national security frame, or no message.^35^

Health framing does not seem to affect the perceived personal importance of climate change, either in pre-post analysis^16^ or in between-group analysis comparing health frame with other frames (eg, national security) and no-message control.^16^^,^^35^ Other cognitive outcomes, such as belief in climate change,^57^ perceived personal responsibility,^39^ perceived need for societal action,^32^ and perceived urgency of addressing climate change,^32^ were reported in single studies and hence could not be synthesized.

In summary, evidence consistently shows that health-framed environmental messages increase threat perception, outperforming environment and economic frames, particularly when these messages are personally relevant. However, this effect may be diminished when messages are overly catastrophic. Evidence of health framing effects on other cognitive responses is either mixed or limited.

#### Policy attitudes

Policy attitudes were assessed in 21 studies,^9^^,^^10^^,^^17^^,^^18^^,^^25–27^^,^^32^^,^^33^^,^^37^^,^^39^^,^^41–44^^,^^47^^,^^49^^,^^53^^,^^56^^,^^57^ with most measuring support for government action (eg, whether the government should do more to support clean energy use) or support for specific policies (eg, natural gas ban). Most studies used between-group designs to compare different message frames (19 studies), while 2 adopted within-group designs.^9^^,^^49^

A pre-post study with 1644 adults found that after reading about the health consequences of air pollution, participants showed decreased support for fossil fuel use, increased opposition to new fossil fuel power plants, and greater support for clean energy and government efforts.^49^ In another within-subject study of 7500 adults, participants were presented with pairs of climate messages with different frames and asked to select the one that elicited greater support for climate policies.^9^ It was found that health-framed climate messages increased policy support compared to climate messages with environment, economic, or migration frames.^9^

Five between-group studies compared policy attitudes between those who read health-framed climate messages and those who received non-climate–related messages or no message,^17^^,^^27^^,^^41^^,^^44^^,^^53^ with 4 showing greater support for government actions and climate policies in the health frame group.^27^^,^^41^^,^^44^^,^^53^ Nine between-group studies compared health and environment frames, with 4 reporting no difference in policy support,^10^^,^^39^^,^^42^^,^^43^ and 5 reporting higher support among those exposed to the health frame.^18^^,^^25^^,^^26^^,^^33^^,^^47^ Moreover, 3 between-group studies consistently showed that health-framed climate messages outperformed economic frames in boosting policy support.^25^^,^^41^^,^^47^ Limited data on comparisons with other frames (eg, social norm, patriotism) precluded synthesis. Notably, when using a health frame in climate messaging, a between-group study showed that mentioning opponents to climate action (eg, fossil fuel chief executive officers, politicians) and describing their actions to hide the truth about climate change significantly increased support for mitigation policies.^32^

In summary, health framing in environmental communication consistently boosts support for government actions and environmental policies. Data also suggest that health frames are at least as effective, and sometimes even more so, in increasing policy support compared to environment and economic frames.

#### Behavioral outcomes

Behavior was a highly heterogeneous group of outcomes assessed in 24 studies.^10^^,^^17^^,^^19–21^^,^^23^^,^^24^^,^^29^^,^^31–33^^,^^35^^,^^37–40^^,^^43^^,^^46^^,^^48^^,^^49^^,^^54^ The assessed behaviors are roughly divided into 2 categories: (1) sustainable lifestyle behavior, such as using reusable shopping bags and commuting by public transit^10^^,^^35^^,^^39^^,^^54^; (2) climate advocacy behavior, such as signing petitions and joining environmental groups.^10^^,^^19–21^^,^^23^^,^^31–33^^,^^35^^,^^37^^,^^39^^,^^43^^,^^49^; and (3) health-protective behavior, such as checking air quality and closing windows during air pollution events.^17^^,^^38^^,^^40^^,^^46^^,^^48^ Some studies combined sustainable lifestyle and climate advocacy behaviors into one outcome (ie, climate mitigation behavior).^24^^,^^29^ Only 10 studies measured actual behavior,^19–21^^,^^32^^,^^33^^,^^37^^,^^38^^,^^48^ with the rest assessing behavioral intentions.

Sustainable lifestyle behavior was assessed in 6 studies,^10^^,^^24^^,^^29^^,^^35^^,^^39^^,^^54^ all of which measured intentions, and none found significant effects of health framing compared to environment framing, national security framing, or no message.

In terms of climate advocacy behavioral intentions, findings were mixed. Although in quantitative non-randomized studies and qualitative studies, advocacy intentions increased following exposure to climate health messages,^23^^,^^31^^,^^49^ RCTs generally found no difference between health framing and environment or national security framing, or no message.^10^^,^^24^^,^^35^^,^^39^^,^^43^ In addition, there is evidence that emphasizing climate solutions and their health benefits, and explicitly mentioning opponents in climate health messages, may enhance advocacy intentions.^31^^,^^32^

Eight studies measured actual advocacy behaviors,^19–21^^,^^32^^,^^33^^,^^37^ with mixed results. An online study presented participants with a set of statements each emphasizing one benefit of climate mitigation (ie, health, environment, competence, communality, development) and asked participants to reproduce from memory and share the messages on social media.^21^ It was found that statements related to health were more likely to be re-posted than statements emphasizing competence, communality, and development, but there was no difference between health and environment statements in the tendency to re-post.^21^ Similarly, a real-world experiment involving 335 members of Canadian Parliament found no evidence that a health frame is more effective than an environment frame in eliciting pro-climate posts on Twitter.^19^ Four RCTs assessed participants’ likelihood of completing advocacy actions (eg, clicking a link to sign a petition); 2 of these found no difference between those who read a climate health message, a non-health–related climate message, and those who received no message,^32^^,^^37^ whereas 2 others reported that climate health messages decreased participants’ tendency to take action compared to standard climate messages.^33^ Furthermore, including normative information in climate health messages, explicitly mentioning opponents to climate action, or using gain versus loss frames, did not seem to influence actual advocacy behavior.^20^^,^^32^^,^^37^

Finally, health-protective behavior was measured in 6 RCTs.^17^^,^^38^^,^^40^^,^^46^^,^^48^ Five of these evaluated messages about the health implications of air pollution.^38^^,^^40^^,^^46^^,^^48^ Overall, these studies consistently showed that health-framed air pollution messages increased both intention and actual protective behaviors against air pollution, compared to standard air pollution messages without a health frame, or no-message control conditions.^38^^,^^46^^,^^48^ Moreover, when communicating the health relevance of air pollution, describing the easy precautions one could take to protect themselves (ie, high-efficacy) significantly increased precaution intention compared to just stating health impacts (ie, low-efficacy).^40^ In addition, 1 study exposed participants to messages describing the risk of contracting dengue and how that risk will increase given climate change.^17^ Participants in this study who read the message emphasizing public health risks, but not personal health risks, had significantly greater intention to receive a hypothetical dengue fever vaccine compared to those who read a control message not related to climate change or health.^17^

In summary, health-framed environmental messages generally failed to influence intentions for sustainable lifestyle changes, with no study assessing actual behaviors. Similarly, there is a lack of consistent and strong evidence supporting the effects of climate health messages on climate advocacy intentions or actions. However, these messages increased health-protective intentions and behaviors, particularly when practical advice was provided.

#### Moderators and mediators

Some studies identified differential impacts of health-framed environmental message on subgroups of participants, despite no significant overall effects. The most commonly assessed moderators included climate change perceptions, political factors, and health status.

Climate change perceptions included various concepts such as climate awareness, skepticism, and concern. Across quantitative studies, limited evidence suggested that health framing effects varied with these perceptions.^9^^,^^10^^,^^17^^,^^26^ However, a qualitative study found that participants with high climate concern responded more positively to health-framed climate messages and sought more information after reading, whereas those with low concern were more negative and critical, and remained skeptical after reading.^11^ One study using the Global Warming’s Six Americas framework found that the Alarmed and Concerned audience segments responded positively to a health-framed climate essay, whereas the Cautious, Disengaged, and Doubtful segments showed less consistency in their responses.^34^ Another study using this framework found that while a public health frame may elicit emotional reactions consistent with support for climate action (ie, hope) across segments, a national security frame may elicit unintended negative reactions (ie, anger) among the Doubtful and Dismissive segments.^36^

Many studies also explored the moderating effects of political factors, including political ideology (eg, conservative, moderate, liberal) and party identification (eg, republican, independent, democrat). These studies were all conducted in the United States. Analyses of political ideology yielded mixed results. Some studies found no moderation effect,^10^^,^^21^^,^^30^ while others indicated stronger health framing effects among conservatives.^15^^,^^16^^,^^32^^,^^39^ For instance, Petrovic et al. found that conservatives, but not liberals, were more likely to support climate policies when shown a health-framed air pollution message, compared to an environment-framed message.^39^ This effect was attributed to a ceiling effect where liberals tend to have higher baseline engagement with climate change, leaving little room for further attitudinal change.^15^^,^^16^ Similarly, studies examining party identification as a moderator yielded mixed results. Six studies found no difference in health framing effects across party affiliations.^31–33^^,^^42^^,^^49^ However, in 2 between-group studies, emphasizing the health benefits of climate policies increased policy support among republicans, but not democrats.^22^^,^^44^

Although less frequently assessed in the included studies, health status emerged as another key moderator with consistent findings. Two studies found that people with poorer health had more negative emotions, greater perceived threat, and stronger policy support after reading messages about climate change and health impacts, compared to people with better health.^15^^,^^16^ Two other studies found that people with asthma were more likely to check air quality after reading health-framed air pollution messages compared to those without asthma.^38^

Finally, indirect effects were explored in some studies. For instance, one study showed that emphasizing the health benefits of low-carbon energy policies boosted support through enhancing the perceived benefits.^22^ In another study, emphasizing the health impacts of air pollution increased perceived threat, which in turn contributed to greater health-protective intentions.^46^

## Discussion

This mixed-methods systematic review synthesizes evidence on health framing in text-based environmental communication. Despite heterogeneity in study designs, message content, and outcome measures, findings indicate that health-framed messages about environmental issues are generally seen as clear and helpful, particularly when employing a gain frame that emphasizes the health benefits of mitigation. There is consistent evidence that health framing can increase perceived threat of environmental issues, strengthen support for environmental policies, and may motivate health-protective behaviors. These messages also appear to engage diverse audiences. Individuals with poorer health who are often disproportionately affected by GECs responded favorably to such messages. Additionally, the observed effects were somewhat larger among groups typically less engaged with GEC issues, such as those with more conservative political orientations. While this pattern has led some authors to suggest that health framing is less politically polarizing than conventional climate messaging, current evidence remains mixed and too sparse to conclude that health framing reduces political polarization around the issue of GEC.

Despite these promising findings, the effects of health framing on sustainable lifestyle changes and advocacy behaviors are less consistent. Although many studies noted increased policy support after reading health-framed messages, these attitudinal shifts did not consistently translate into individual-level behavioral change. This divergence highlights a potential limitation: while health framing may enhance personal salience of climate risks (ie, “how does this affect me”) and motivate self-protective intentions (ie, “what can I do to protect myself”), it may be less effective in promoting behaviors that are altruistic, collective, or require sustained personal investment. One possible explanation is that when the perceived efficacy of individual action is low, health-framed messages alone may be insufficient to motivate broader mitigation behaviors. Thus, health framing may be more successful in influencing self-directed outcomes than in catalyzing pro-environmental behaviors aimed at societal benefits or planetary health.

It is also important to note that health framing effects may vary along several dimensions of the target behavior: emissions-reduction potential (eg, how much a behavior reduces emissions), immediacy of health co-benefits (eg, how soon a behavior improves health), and plasticity (eg, how easily people can adopt a new behavior). Climate advocacy actions such as voting can drive large-scale climate impact, yet their personal health benefits are often distant and abstract, providing limited individual incentive for change.^58^ In contrast, health-protective behaviors such as wearing a mask and running an air purifier during air pollution events, yield more tangible and immediate health gains and hence may be easier to prompt, but the extra energy usage and waste production can undermine mitigation. It is possible that health-framed messages may work best for behaviors that deliver quick, visible health benefits, irrespective of their environmental footprint, a hypothesis that aligns with our findings but warrants further direct testing. Future work should map outcomes onto these dimensions and pre-specify the category and impact level of the behavior being targeted before selecting a health frame.

Interestingly, 2 studies in this review noted that health-framed messages, particularly those emphasizing personal health risks of climate change, decreased rather than increased climate advocacy behavior compared to standard climate messages.^33^ One possible explanation is that a strong or narrow focus on personal, rather than public health impacts, may be perceived as overly threatening, which could unintentionally lead to defensive or avoidant reactions, as consistently shown in the fear appeal literature.^59^ More work is clearly needed, as most studies in this review evaluated messages that framed GECs as a public health issue. Very little research has directly tested whether framing GECs in terms of local and personal impacts or in terms of societal and global impacts is more effective in engaging the audiences. One study found that framing environmental health risks as more personally relevant and geographically proximate increased perceived threat,^52^ while a large international experimental study showed that a global frame of climate change increased policy support, whereas an individual frame reduced support.^9^ Similarly, another large experimental study found that emphasizing public health risks of climate change, but not personal health risks, increased health-protective intention.^17^ Taken together, these findings highlight a critical gap in the evidence base: whether health framing is more effective when presented at an individual, community, national, or global scale. Future studies should take a more nuanced approach to examine how different levels of health framing affect engagement, motivation, and sustained pro-environmental actions.

This review offers several practical implications. First, the findings indicate that adopting a health frame in environmental communication can effectively engage diverse audiences on the issue of GECs and can increase threat perception, climate policy support, and health-protective behaviors. By making environmental issues more relatable, health framing in public education and policy communication about GECs may shift attitudes and behaviors at the population level, contributing to both human and planetary health outcomes. Another implication is the unique role of health professionals as trusted messengers in this space. Their clinical credibility, public-facing roles, and frequent interactions with both the general and vulnerable populations make them well-positioned to convey the health risks of GECs and advocate for mitigation.^5^ As such, healthcare settings offer a particularly relevant yet underutilized platform to integrate environmental communication, such as through climate-sensitive health counseling.^60^

Based on the synthesized evidence, we summarized some key recommendations for future communication strategies and future research, which are detailed in Table 3. A key future direction is adopting a user-centered approach to design environmental health messages. Co-design, a collaborative process involving service users and key relevant parties, has been shown to improve service quality and user engagement by aligning with target populations’ experiences, needs, priorities, and values.^61^ In this review, most messages were created by researchers without input from audiences. Increasingly used in health communication, co-design has been successfully applied in contexts such as cancer recovery and obesity prevention, resulting in highly-rated health message banks.^62^^,^^63^ Similar efforts in GEC communication will likely enhance the acceptability and effectiveness of health-framed messages, ensuring that the messages resonate with diverse audiences and address their specific needs and concerns.

**Table 3:** Evidence-based recommendations.

Future text-based communication strategies	
**Use a threat-efficacy structure by highlighting the health threat of environmental issues followed by the health benefits of mitigation and adaptation strategies**	
**Utilize gain framing to emphasize health benefits**	
**Provide easy precautions one could take to reduce the risk of adverse health outcomes that result from environmental issues**	
**Include stories and lived experiences**	
**Use statistics with references to credible sources**	
**Tailor messages to the target audience to address specific concerns**	
**Future research**	
**Expand research efforts to low- and middle-income countries, and to vulnerable individuals**	
**Report randomization and blinding procedures in randomized controlled trials**	
**Ensure baseline comparability between groups in randomized controlled trials**	
**Perform manipulation or attention check after message exposure to confirm adherence to interventions**	
**Explore health framing effects in contexts beyond climate change and air pollution**	
**Examine the effectiveness of health framing at different levels, from individual or community to national or global scale**	
**Investigate when a health frame is the most effective, for whom, from which messenger, and in what context**	
**Use participatory research methods to co-create messages with input from target audience and relevant parties**	
**Incorporate ecologically valid outcome measures that assess actual behavior, not just intentions or attitudes**	
**Understand the mechanisms underlying health framing effects through exploring moderators and mediators**	
**Explore the effectiveness of multi-media communication strategies beyond text-based messages**	
**Evaluate if multi-frame messaging strategies are more effective than health-only frames**	

Second, while the present review adopted a comprehensive approach to include health messages linked to any aspect of GECs, health framing effects may vary across environmental domains as the target behaviors in each domain are distinct and likely carry different cultural meanings. Consequently, health communication strategies optimized for climate change may not directly generalize to other GECs such as biodiversity loss. Future studies should co-develop and test health-framed messages that are explicitly tailored to the specific environmental issue, rather than extrapolating from climate communication paradigms.

Furthermore, although the focus on text messages in this review controls for confounding effects from other media, real-world audiences include visual learners and low-literacy users who may organize information spatially. Future work should test whether embedding health-framed messages within infographics, videos, or other mediums enhances attitudinal and behavioral change.

Finally, future research should examine how health-framed messages operate alongside other frames such as those highlighting economic, environmental, or social-normative aspects, and how their combined influence is moderated by messenger credibility and recipients’ political, religious, or cultural identities. Integrating complementary frames can extend reach to audience segments less responsive to health-only narratives. A multi-frame strategy may likely produce stronger effects across diverse populations.

This review has several limitations. First, many studies lacked details on randomization and blinding, potentially affecting the reliability of findings. Second, although we did not exclude studies based on language, all included articles were published in English, with the vast majority conducted in high-income countries, limiting generalizability and highlighting a need for future research in low- and middle-income countries. Third, most studies involved only a single exposure to health-framed environmental messages, with outcomes assessed immediately afterward. This approach may not allow sufficient time for attitudinal changes to translate into actual behaviors. Future studies should adopt longitudinal designs with longer follow-up assessments and evaluate the effects of repeated message exposure over time. This will help determine whether and how health-framed messages can lead to sustained and meaningful behavioral changes. In addition, most studies focused on the general public, with few on vulnerable groups like the elderly. Finally, the lack of available data and heterogeneity of study methodology precluded a quantitative synthesis, limiting our ability to conclude on the size of observed effects.

Despite these limitations, this review provides a comprehensive synthesis of extant research on the health framing effects in text-based environmental communication. The findings highlight the potential of health framing to enhance threat perception, boost policy support, and motivate health-protective behaviors, particularly among individuals with poorer health and those who tend to be dismissive of the issue of GECs. Future research should incorporate rigorous designs, more diverse populations, co-design methodologies, and focus on long-term, real-world outcomes to develop more impactful communication strategies at the intersection of GECs and health. By addressing these gaps, future studies can provide clearer guidance on effective communication strategies in the face of impending environmental and health crises.

## Supplementary Material

kaag002_Supplementary_Data

## Transparency statements

- The study was pre-registered at PROSPERO (CRD420251050978).
- The analysis plan was registered prior to beginning data collection at PROSPERO (CRD420251050978).
- De-identified data from this study are not available in a public archive. De-identified data from this study will be made available (as allowable according to institutional IRB standards) by emailing the corresponding author.
- There is no analytic code associated with this study.
- Materials used to conduct the study are not publicly available.
