# Supplementary material for: Communicating the health implications of global environmental change: a mixed-methods systematic review of health framing in environmental messaging
Source: Ann Behav Med. 2026 Feb 12;60(1):kaag002. doi: 10.1093/abm/kaag002 (PMC13017760; doi:10.1093/abm/kaag002)
Supplement: kaag002_Supplementary_Data [file kaag002_supplementary_data.docx]

**Electronic Supplementary Materials**

***Electronic Supplementary Material 1*: Sample electronic database search strategy (PubMed)**

|  | **Search Terms** | **Results (16 Aug 2024)** |
| --- | --- | --- |
| #1 | "Climate Change"[MeSH Terms] OR "Environmental Pollution"[MeSH Terms] | 686923 |
| #2 | “environmental change”[Title/Abstract] OR “environmental damag*”[Title/Abstract] OR “environmental harm”[Title/Abstract] OR “environmental policy”[Title/Abstract] OR “climate change”[Title/Abstract] OR “climatic change”[Title/Abstract] OR “global warming”[Title/Abstract] OR “extreme weather”[Title/Abstract] OR heat[Title/Abstract] OR pollution[Title/Abstract] OR “fossil fuel*”[Title/Abstract] OR “carbon emission*”[Title/Abstract] OR “greenhouse gas emission*”[Title/Abstract] OR “biodiversity loss”[Title/Abstract] OR sustainability[Title/Abstract] OR planetary [Title/Abstract] | 555755 |
| #3 | framing[Title/Abstract] OR frame[Title/Abstract] OR framed[Title/Abstract] OR refram*[Title/Abstract] OR messag*[Title/Abstract] OR text[Title/Abstract] OR essay*[Title/Abstract] OR news[Title/Abstract] OR “information intervention”[Title/Abstract] OR “information treatment”[Title/Abstract] | 392239 |
| #4 | health[Title/Abstract] OR co-benefit[Title/Abstract] | 2833444 |
| #5 | #1 OR #2 | 1120464 |
| #6 | #3 AND #4 AND #5 | 3103 |

***Electronic Supplementary Material 2*: Study characteristics and full reference list**

| **Author, year** | **Study objective** | **Study location** | **Study design** | **Participant** | **Message intervention** | **Comparison** | **Outcome** |
| --- | --- | --- | --- | --- | --- | --- | --- |
| Bernauer & McGrath, 2016 (Study 2) | To examine if reframing climate mitigation policies from "an effort to reduce or avoid climate change risks" to "an effort to protect the public from climate-change-induced health hazards", could increase public's support for climate policies. | United States | Quantitative randomised controlled trial | Adults; N = 672; Female: 37·0%; Age range: 18-76; Mean age = 30·8 | Participants read a message about climate mitigation strategies (150-250 words) emphasising either climate risks or health benefits. | Climate risk frame (emphasised risks and potential catastrophes that failure to combat climate change would lead to) vs. Health benefits frame (emphasised how combating climate change would improve health through pollution reduction and encouraging more active lifestyles) | Policy attitudes regarding climate change mitigation; Behavioural change intentions; Environmental citizenship intentions |
| Buchanan et al., 2022 | To assess if support for action and policies at COP26 is influenced by how the topic is framed. | International | Quantitative randomised controlled trial | Adults; N = 14627 (about 2000 per country); Female: 49·7%; Age range: 18+ | All participants read a message of roughly 230-250 words. Participants in the control group read a message that made no reference to climate change. Other subjects read a message about climate change and mitigation strategies emphasising (1) the current UN messaging, (2) public health, (3) social norm, or (4) patriotism. | Control vs. Current UN messaging (emphasised the science around climate change and urgency for action) vs. Public health frame (focused on the need to reduce pollution and the health impacts of climate change) vs. Social norms frame (mentioned the scientific consensus that human-caused climate change is underway and reported data demonstrating the public's common belief in human-caused climate change) vs. Patriotism (included reference to a local species at risk from climate change and emphasised the duty to protect the land and children) | Support for government action; Support for policies |
| Cameron et al., 2021 | To explore public attitudes regarding communication tools focused on climate change and climate-affected Lyme disease. | Canada | Qualitative study | Adults from three different communities; N = 61; Age range: 21-80; Mean age = 51·0 | Participants read a 1200-word plain language article about climate change and climate-affected Lyme disease. | N/A | Evaluation of the message |
| Campbell, 2023 (Study 1) | To examine if different types of social normative appeals can help engage health professionals in climate advocacy. | International | Quantitative randomised controlled trial | Health professionals; N = 211 | Participants read a message (around 50 words) encouraging them to sign up to receive information about opportunities to advocate for actions to protect the public's health from climate change. | Control condition (message without normative information) vs. Descriptive norm condition (message with descriptive normative information) vs. Dynamic norm condition (message with dynamic normative information) | Provision of email address to sign up and receive information |
| Campbell, 2023 (Study 2) | To examine if different types of social normative appeals can help engage health professionals in climate advocacy. | International | Quantitative randomised controlled trial | Health professionals; N = 8323 | All participants received an email (about 250 words) about the health harms of fossil fuels and a treaty to stop fossil fuels. | Control condition (message without normative information) vs. Descriptive norm condition (message with descriptive normative information) vs. Descriptive + dynamic norm condition (message with both descriptive and dynamic normative information) | Completion of an advocacy action via embedded links |
| Connor et al., 2016 | To examine what types of climate change messages survive longer when passed between individuals via communication network chains. | United States | Quantitative randomised controlled trial | Facebook-using adults; N = 207; Female: 45·4%; Age range: 18-67; Mean age = 32·3 | Participants read a message about climate change mitigation formatted like a Facebook post and were asked to imagine it was posted by a Facebook friend. All messages contained 10 statements, two from each content domain: nature, competence, communality, development, and health. Participants were randomly assigned to the (1) all gain, (2) all loss, or (3) mixed condition. | Between-subject comparison: All gain frame (benefits of climate change mitigation) vs. All loss frame (costs of non-mitigation) vs. Mixed frame (one gain-framed and one loss-framed statement from each content domain);  Within-subject comparison: Nature frame (how climate change will affect plants, animals, and the likelihood of natural disasters) vs. Competence frame (how climate actions allow us to learn new skills and advance scientific knowledge) vs. Communality frame (how caring and warm we are to each other) vs. Development frame (relating climate change to the economy and technology) vs. Health frame (linking climate change to infectious diseases and the effects of airborne contaminants) | Subjects were asked to repost the statements read in their own words. The messages that were reproduced by participants first in the chain were then presented to participants second in the chain, and the reproductions from participants second in the chain were presented to participants third in the chain. Statements were then coded into reproduced, abstracted, new statements, or disagreements. |
| Dasandi et al., 2022 | To test the effects of four different types of frames, or attributes, on support for climate change policies. | International | Quantitative non-randomised study | Adults; N = 7512 (about 1500 per country); Female: 50·3%; Age range: 18+ | Each participant was shown five pairs of two statements about climate change and mitigation strategies displayed side by side. The statements consisted of four attributes: valence, theme, scale, and timeframe. The four attributes were varied in the statements provided to participants. | Valence: Positive frame (emphasised the opportunities that tackling climate change provides) vs. Negative frame (highlighted the threats that climate change poses);  Theme: Economic frame vs. Environmental frame vs. Health frame vs. Migration frame;  Scale: Personal level vs. Community level vs. National level vs. Global level;  Timeframe: Present vs. 2030 vs. 2050 | Selection of messages which made participants more likely to support policies to tackle climate change |
| Delmas & Kohli, 2021 (Study 1) | To study people's engagement with real-time air quality information and to determine how to effectively frame air quality information to promote public health. | United States | Quantitative randomised controlled trial | Adults; N = 835; Female: 43·0%; Mean age = 35·3 | Participants read a message (within 40 words) about the health impacts of air pollution with different emphasis (exercise, child asthma, child cognition, Alzheimer's, air pollution invisibility) and valence (positive, negative, mixed). | Emphasis: Control (no specific health impact) vs. Exercise (health effects of air pollution when exercising outdoors) vs. Child asthma (effects of air pollution on childhood asthma); Child cognition (effects of air pollution on brain development); Alzheimer's (effects of air pollution on Alzheimer's disease); Air pollution invisibility (highlighted that even if air pollution is invisible, it can still have adverse health effects)  Valence: Positive frame (emphasised health benefits of avoiding air pollution) vs. Negative frame (emphasised health risks of air pollution) vs. Mixed (combined positive and negative frames) | Message comprehensibility; Message realism; Message relevance; App engagement intention |
| Delmas & Kohli, 2021 (Study 2) | To study people's engagement with real-time air quality information and to determine how to effectively frame air quality information to promote public health. | United States | Quantitative randomised controlled trial | Adults; N = 2740; Female: 44·7%; Age range: 18+ | Participants were randomly assigned to different message conditions and received one message per week via email for five consecutive weeks. An additional control group received no email. | Emphasis: Control (no specific health impact) vs. Exercise (health effects of air pollution when exercising outdoors) vs. Child asthma (effects of air pollution on childhood asthma); Child cognition (effects of air pollution on brain development); Alzheimer's (effects of air pollution on Alzheimer's disease); Air pollution invisibility (highlighted that even if air pollution is invisible, it can still have adverse health effects)  Valence: Positive frame (emphasised health benefits of avoiding air pollution) vs. Negative frame (emphasised health risks of air pollution) vs. Mixed (combined positive and negative frames) | Weekly app visits |
| Feldman & Sol Hart, 2018a (Study 1) | To test the effects of six different climate change frames on selective exposure to climate change news. | United States | Quantitative non-randomised study | Adults; N = 600; Female: 51·0%; Mean age = 45·1 | Participants were shown the headlines and leads for six news stories about climate change and mitigation strategies, each with a different frame (conflict, economic, environmental, moral, national security, and public health). The stories were formatted to resemble the first six results from a Google News search. Participants were asked browse through the articles and were encouraged to read what they find interesting. They were told that “the scheduled time does not allow for reading all articles,” thus encouraging them to be selective. After clicking on the headline of the story they wanted to read, they were taken to the full story text. After reading, they were able to return to the headline page to make another article choice if desired. | Conflict (emphasised political conflict over whether and how to address climate change) vs. Economic (focused on economic implications of climate change and of mitigating climate change in terms of jobs, investments, markets, industry) vs. Environmental (emphasised impacts on ecosystems and animal species, including extreme weather, sea level rise, ice melt, coral reef degradation, etc.) vs. Moral (includes an explicit moral, religious or ethical call to act on climate change) vs. National security (emphasised threat of climate change to human security, particularly as a result of violent conflict and human migration) vs. Public health (emphasised human health impacts of climate change, including asthma, allergies, infectious disease, malnutrition, etc.) | Time spent reading each article |
| Feldman & Sol Hart, 2018a (Study 2) | To test the effects of six different climate change frames on selective exposure to climate change news. | United States | Quantitative randomised controlled trial | Adults; N = 1466; Female: 51·0%; Mean age = 45·5 | Participants were shown the headlines and leads for six news stories (one about climate change amid five on other news topics). Participants were randomly assigned to see a climate change story using one of the six frames (conflict, economic, environmental, moral, national security, and public health). Procedures are similar to that of Study 1. | Conflict (emphasised political conflict over whether and how to address climate change) vs. Economic (focused on economic implications of climate change and of mitigating climate change in terms of jobs, investments, markets, industry) vs. Environmental (emphasised impacts on ecosystems and animal species, including extreme weather, sea level rise, ice melt, coral reef degradation, etc.) vs. Moral (includes an explicit moral, religious or ethical call to act on climate change) vs. National security (emphasised threat of climate change to human security, particularly as a result of violent conflict and human migration) vs. Public health (emphasised human health impacts of climate change, including asthma, allergies, infectious disease, malnutrition, etc.) | Time spent reading each article |
| Feldman & Sol Hart, 2018b | To examine how public support for four specific low-carbon energy policies varies when these policies are framed as a way to reduce either climate change, air pollution, or energy dependence. | United States | Quantitative randomised controlled trial | N = 1000; Female: 54%; Mean age = 47·4 | Participants were shown several possible low-carbon energy policies proposed by legislators (about 30 words). | Climate change condition (described the policies as a way to help reduce greenhouse gas emissions and stop climate change) vs. Air pollution condition (described the policies as a way to help reduce air pollution and its related health effects) vs. Energy security condition (described the policies as a way to help reduce dependence on foreign sources of oil) | Support for the policies; Perceived benefits and costs of policies |
| Godawska, 2020 | To examine how alternative ways of framing sulphur dioxide pollution problem (highlighting consequences for human health, nature and state finance) affect the public support for abatement policy (emission fees and emission trading) and bearing higher heating costs. | Poland | Quantitative randomised controlled trial | University students; N = 288; Socio-demographic data not collected | Participants read a short description (60-120 words) of the consequences of excessive sulphur dioxide emission, presented in different frames referring to human health, nature, and financial penalties. | Human health frame (emphasised how sulphur dioxide is harmful to human health) vs. Nature frame (emphasised how sulphur dioxide is harmful to plants and forests) vs. Financial penalties (emphasised how sulphur dioxide emission will incur national financial penalty) | Support for (1) the imposition of additional emission fees, (2) launching of the cap-and-trade program, and (3) voluntarily bearing higher heating costs due to the use of the least sulphur-containing fuel |
| Hubbert et al., 2020 | To evaluate how hospital staff react to the proposition that sustainability solutions are also health solutions. | United States | Qualitative study | Hospital staff; N = 16 | Participants read a one-page essay (488 words) that described sustainability solutions as health solutions, including brief descriptions of six categories of solutions that could be implemented in their hospital system (energy, food, transportation, buildings, consumption, and actions that can be taken in surrounding cities and communities). | Each sentence in the essay | General reaction to the essay; Passages of the essay that they liked or found to be particularly helpful and those they did not like or found unhelpful |
| Jasemzadeh et al., 2018 | To investigate an extended parallel process model-based mobile phone text message intervention for improving protective behaviours against air pollution among pregnant women. | Iran | Quantitative randomised controlled trial | Pregnant women; N = 125; Mean age = 26·8 | Two to seven short messages about the health effects of air pollution on pregnancy outcomes and recommended behaviour to avoid the health risks due to air pollution were sent to participants in the intervention group over two months. | Control group (received no message) vs. Intervention group | Perceived susceptibility; Perceived severity; Response efficacy; Self-efficacy; Air pollution protective behaviours |
| Joshi, 2022 | To examine whether framing the risk of biodiversity loss in terms of its impacts on human health can persuade people to adopt recommended sustainable behaviours to protect themselves against its harmful effects on their health. | United States | Quantitative randomised controlled trial | Adults; N = 676; Female: 51·8%; Age range: 18+ | Participants read either a high-risk or a low-risk message about the risks posed by biodiversity loss to human health. Both messages used the same factual information. The only difference was that the health risks were emphasised in the high-risk message, whereas the low-risk message was not as catastrophic. | High-risk frame vs. Low-risk frame | Threat appraisal; Fear; Protection motivation (intention to adopt a recommended response) |
| Kim et al., 2021 | To investigate whether cognitive and emotional appraisals of messages about climate change related health risks would mediate the relationships between participants' individual differences (in political ideology and health) and their perceived harm to self and support for climate change policies. | United States | Quantitative non-randomised study | Adults; N = 1104; Female: 54·0%; Age: 48·7% under 44 | All participants read eight essays (140-180 words each) about different categories of health impacts from climate change. The essays were structured based on three core questions: “What is happening?”; “How does that harm our health?”; and “Who is being harmed?” Different content was presented in each message based on the given health impact category, including extreme heat, poor air quality, extreme weather events, diseases spread by insects, ticks and rodents, contaminated water, contaminated food, hunger and malnutrition, and mental health problems. | Pretest vs. Immediate posttest vs. 2-week posttest | Cognitive appraisal of messages; Emotional appraisal of messages; Perceived harm to self; Policy support |
| Kotcher et al., 2018 | To examine how Americans react to information about eight specific categories of health impacts from global warming. | United States | Quantitative randomised controlled trial | Adults; N = 2254; Female: 53·4%; Age range: 18+ | Participants in the intervention group read eight essays (140-180 words each) about different categories of health impacts from climate change. The essays were structured based on three core questions: “What is happening?”; “How does that harm our health?”; and “Who is being harmed?” Different content was presented in each message based on the given health impact category, including extreme heat, poor air quality, extreme weather events, diseases spread by insects, ticks and rodents, contaminated water, contaminated food, hunger and malnutrition, and mental health problems. | Control group (received no information) vs. Intervention group | Global warming beliefs: Affective assessment of the health impact of global warming; Affective assessment certainty; Personal issue importance; Worry about global warming; Perceived harm to self; Perceived harm to future generations; Injunctive beliefs;  Essay evaluation: Perceived relevance; Perceived novelty of the information; Ease of understanding the information; Negative emotional response; Timing of the impacts |
| Kotcher et al., 2019 | To identify specific messages about the health implications of air pollution from fossil fuels that are most and least concerning to people, and to test whether exposure to these messages influence people's attitudes and behavioural intentions. | United States | Quantitative non-randomised study | Adults; N = 1644 (396 older adults, 334 with low income, 465 mothers of young children, 150 expectant mothers, 201 childcare providers, 288 healthcare professionals); Female: 65·3%; Age range: 18+ | Participants engaged in a maximum difference scaling exercise to elicit their ranking of ten different statements (20-40 words) about the health consequences of air pollution caused by burning fossil fuels. The statements included well-established health harms such as asthma, cancer, and heart disease; emerging neurological health harms to children; emerging neurological health harms to older adults; mechanisms by which air pollution causes harm to health; and statements about who is most likely to be harmed by air pollution from fossil fuels. The ten statements were shown to each respondent multiple times across eight screens, with each screen displaying a different combination of four statements. | Ten statements | Ranking of statements that cause the most and least concern; Perceived health risk of air pollution; Perceived health harm from fossil fuels; Support for fossil fuel energy use; Support for new fossil fuel plant near one's home; Support for clean energy use; Support for government and industry leadership on clean energy; Consumer advocacy intentions; Political advocacy intentions |
| Kotcher et. al, 2021 | To test the motivational value of three categories of climate information: health consequences of climate change; health benefits of climate solutions; and calls-to-action intended to motivate people to engage in political advocacy for climate solutions. | United States | Quantitative non-randomised study | Adults; N = 7596; Female: 52·7% | On each screen, participants were presented two messages side by side. Every participant evaluated four pairs of randomly selected messages presented across multiple screens. The messages all had the same basic structure, beginning with a section titled “The Problem” that briefly described one type of health harm associated with climate change; followed by a second section titled “The Solution” that described a type of climate solution and its health benefits; and concluding with a section titled “How you can help” that included a call to action which specifically asked participants to contact their member of Congress and urge them to address this issue. | "The problem": Control (no information on health impact) vs. Extreme heat vs. Poor air quality vs. Extreme weather vs. Water-borne disease vs. Food-borne disease vs. Vector-borne disease vs. Hunger and malnutrition vs. Mental health;  "The solution": Control (no information on solution) vs. Sustainable energy vs. Cities and communities vs. Consumption vs. Nature spaces vs. Transportation vs. Buildings vs. Food;  "Call to action": Control (no call to action) vs. Positive descriptive norms vs. Negative descriptive norms vs. Surplus efficacy vs. Deficit efficacy | Selection of messages that would make participants most likely to contact their member of Congress |
| Kotcher et. al, 2023 | Previous research suggests that providing information about the health effects of climate change and the health benefits of climate action can increase public engagement with the issue. This study extends these findings with an experiment to test the motivational value of calling attention to opponents of climate action. | United States | Quantitative randomised controlled trial | Adults; N = 2201; Female: 53·0%; Age range: 18+ | Participants in the no-message control group were not shown any message. All other participants read a message (280-390 words) about the health harms caused by climate change, followed by information about the health benefits of climate solutions. Participants who read a message were randomly assigned to either the standard message control condition, or one of the three opponent conditions: (1) fossil fuels condition, (2) politicians condition, and (3) combined condition. In the three opponent conditions, the opponent and their actions to hide the truth about climate change were explicitly mentioned. All messages ended with a call to action. | No-message control vs. Standard message control (message without portrayal of a climate actions opponent) vs. Fossil fuels condition (message with a portrayal of fossil fuel CEOs and their lobbyists as the opponent) vs. Politicians condition (message with a portrayal of some politicians as the opponent) vs. Combined condition (message with a portrayal of fossil fuel CEOs and their lobbyists, and the politicians who are in their pockets as the opponent) | Anger; Perceived responsibility of the opponent; Mitigation policy support; Perceived need for societal action; Motivation to take actions; Perceived urgency of addressing climate change; Advocacy behavioural intention; Advocacy behaviour; Trust in health professionals |
| Landrum et al., 2024 | To test whether co-constitutive risk messaging impacts support for pharmaceutical interventions to minimise dengue fever health risks and/or policy efforts to mitigate climate change. | United States | Quantitative randomised controlled trial | Adults; N = 2200; Age range: 18+ | Participants were exposed to one of two co-constitutive risk messages vs. a pure control message pertaining to the history of baseball (200-300 words). Both co-constitutive risk messages emphasised the risk of contracting dengue and how that risk will increase given global climate change. However, the messages varied in cultural cognitive framing, such that one message (“individual risk”) emphasises the spread of mosquito-borne infection as a risk to one’s personal health, while another (“collective risk”) emphasises the public health risks of mosquito-borne infection. | Control message (history of baseball) vs. Individual risk message (emphasised the spread of mosquito-borne infection as a risk to one's personal health) vs. Collective risk message (emphasised the public health risks of mosquito-borne infection) | Support for dengue fever vaccine mandate for children; Prospective vaccination intention; Support for vaccine research and development; Support for renewable energy research and development; Support for greenhouse gas regulation; Support for power plant emission limits; Support for energy source diversification |
| Levine & Kline, 2017 (Study 2) | To evaluate the effect of framing on political engagement on climate change. | United States | Quantitative randomised controlled trial | Women; N = 102556; Age range: 25+ | Subjects were randomly assigned to receive one of two emails. The control group received a short message about climate change which described the problem and then quickly transitioned to efficacy-boosting language about what we have to gain from climate change mitigation policies. The personal health risk group received the same text along with a short passage in the middle that prompted them to think about how climate change would threaten their personal health. | Control group (standard climate message) vs. Personal health risk group (climate message emphasising risk to personal health) | Completion of an advocacy action to sign a petition via an embedded link |
| Levine & Kline, 2017 (Study 3) | To evaluate the effect of framing on attitudinal engagement on climate change. | United States | Quantitative randomised controlled trial | Adults; N = 645; Age range: 18+ | Subjects were randomly assigned to read one of three messages. The control group read a standard message about climate change. The personal health risk group read the same message along with a short passage in the middle that emphasised personal health risk. The food risk group read a message prompting respondents to think about how climate change would threaten their ability to purchase food they need. | Control group (standard climate message) vs. Personal health risk group (climate message emphasising risk to personal health) vs. Food risk group (climate message emphasising risk to food security) | Perceived impact of climate change; Perceived concreteness of climate change; Reality of climate change; Policy attitudes |
| Levine & Kline, 2017 (Study 4) | To evaluate the effect of framing on climate advocacy behaviour. | United States | Quantitative randomised controlled trial | Adults; N = 369; Age range: 18+ | Subjects were randomly assigned to read one of three messages. The control group read a standard message about climate change. The personal health risk group read the same message along with a short passage in the middle that emphasised personal health risk. The food risk group read a message prompting respondents to think about ﻿how climate change would threaten their ability to purchase food they need. | Control group (standard climate message) vs. Personal health risk group (climate message emphasising risk to personal health) vs. Food risk group (climate message emphasising risk to food security) | Completion of an advocacy action to sign up and join a climate advocacy group |
| Li et al., 2022 | To examine how recipients' reactance proneness affects the appraisal of threat and efficacy, which, in turn, influences their use of information-processing modes, attitudes, and behavioural intentions regarding the mitigation of microplastic pollutions in Taiwan. | Taiwan | Quantitative randomised controlled trial | College students; N = 362 (about 120 per condition); Female: 54·0%; Age: 98·0% between 18 and 22 | Participants read a message (around 1000 Chinese characters) about microplastic pollution. Participants were randomly assigned to read (1) a high-threat/high-efficacy message, (2) a high-threat/low-efficacy message, or (3) a low-threat/low-efficacy message. | Threat: High-threat frame (described how microplastics become part of the human food chain and its seriously harmful effect on human health, and reported data showing that 44-61% of Taiwan's tap water contained microplastics) vs. Low-threat frame (used a news story from Italy and described interviews with medical experts who suggested that this is not a serious issue but that if people are concerned, they can install water filters to remove microplastics);  Efficacy: High-efficacy frame (described policies that the Taiwanese government has enacted and the effectiveness of these policies, and offered solutions for individuals to reduce their use of plastics in daily life) vs. Low-efficacy frame (describes the difficulty encountered by the Taiwanese government in reducing people's use of plastic products and provided several solutions that may be difficult for individuals to carry out) | Perceived threat; Perceived efficacy; Information-processing modes; Attitudes toward preventing microplastic pollution; Behavioural intentions toward preventing microplastic pollution |
| Liu et. al, 2021 (Study 2) | To examine the relationship between fear of COVID-19, air pollution concern, and low-carbon behaviours. | Mainland China | Quantitative randomised controlled trial | Adult netizens; N = 304; Female: 45·1%; Age: 71·7% under 35 | Participants read a message (80-90 words) describing the effect of air pollution on infectious diseases such as COVID-19 and efficacy of low carbon behaviours in preventing air pollution and inhibiting spread of these diseases. Participants were randomly assigned to read the message in gain framing or loss framing. | Gain frame (emphasised health benefits of adopting low carbon behaviours) vs. Loss frame (emphasised health risks of not adopting low carbon behaviours) | Air pollution concern; Low carbon behaviours |
| Liu & Yang, 2023 | This study focuses on personal relevance as a key variable that influences risk perception, systematic processing, and information seeking intention. | United States | Quantitative randomised controlled trial | N = 983; Female: 59·5%; Mean age = 51·5 | Participants read a message (120-140 words) about per- and polyfluoroalkyl substances contamination. Participants were randomly assigned to read the message with (1) high personal relevance or (2) low personal relevance. Messages in both conditions mentioned the same health impacts of exposure to per- and polyfluoroalkyl substances. | High personal relevance frame (informed participants about the high level of per- and polyfluoroalkyl substances contamination in their drinking water, based on the zip code they provided, and highlighted the usage of per- and polyfluoroalkyl substances in consumer products such as nonstick cookware and pizza delivery box) vs. Low personal relevance frame (informed participants that their residential area does not have a high level of per- and polyfluoroalkyl substances contamination in drinking water, and mentioned the use of per- and polyfluoroalkyl substances in industrial products such as construction materials and semiconductor) | Risk judgement; Emotional response; Systematic processing; Information seeking intention |
| MacInnis et al., 2015 (Study 2) | To assess whether attitudes toward preparation for the possible effects of global warming varied depending on who endorsed such efforts, the stated purpose of preparation, the consequences of global warming targeted in a preparation message, and the words used to describe preparation and its alternative. | United States | Quantitative randomised controlled trial | Adults; N = 1000; Female: 46·8%; Age range: 18+ | Participants were randomly assigned to hear one of six descriptions of the objectives of climate preparation efforts (reduce risk, reduce vulnerability, increase resilience, increase preparedness, increase readiness, prevent maladaptation), and one of three possible effects of global warming (people and environment, public health, coast and wildlife). | Objectives of preparation efforts: Reduce risk vs. Reduce vulnerability vs. Increase resilience vs. Increase preparedness vs. Increase readiness vs. Prevent maladaptation;  Effects of global warming: ﻿Damage to people, the environment, and property vs. Adverse effects on public health in terms of illnesses and premature deaths vs. Adverse consequences for beaches and property along the coast, seafood supply, and wildlife | Preference for taking steps to prepare for possible consequences of global warming rather than waiting for these consequences to occur and dealing with them then |
| Maibach et al., 2010 | To explore how American adults respond to an essay about climate change framed as a public health issue. | United States | Mixed methods study | Adults; N = 70; Female: 44·3%; Age: 68·6% under 50 | Participants read a brief essay (575 words) designed to frame climate change as a human health issue. The essay was organised into four sections: an opening paragraph that introduced the public health frame; a paragraph that emphasised how human health will be harmed if action is not taken; a paragraph that discussed mitigation-focused policy actions and their health benefits; and a concluding paragraph to reinforce the frame. | Each sentence in the essay | General reaction to the essay; Portion of the essay that were clear or helpful, or were confusing or unhelpful |
| McComas et al., 2015 | To examine the effects of communicating about risks to marine organisms and public health on people's support for policies aimed at mitigating those risks as a function of different message frames. | United States | Quantitative randomised controlled trial | Ferry passengers; N = 543; Female: 58·2%; Mean age = 45·9 | Participants were randomly assigned to one of the five conditions: a no-message control condition and four message conditions. Participants in the message conditions were presented with a fictitious news article (about 190 words) about Vibrio outbreaks and ocean acidification. Depending on the experimental condition, the article highlighted consequences either for public health or oyster health; in addition, effects were attributed either to climate change or global warming. | Consequence: Public health frame vs. Oyster health frame;  Cause: Climate change frame vs. Global warming frame | Concern about marine disease; Support for marine policy to mitigate diseases in the ocean |
| McLean et al., 2024 | To demonstrate how public support for natural gas restrictions varies based on policy framing. | United States | Quantitative randomised controlled trial | Adults; N = 2623; Female: 51·1%; Age: 49·5% under 44 | All participants read a short description of a proposed natural gas ban and then read a set of statements focusing on five policy aspects: (1) economic costs for households, (2) consequences for the gas and renewable energy industries, (3) health effects of natural gas use, (4) climate change implications of natural gas use, and (5) political support for the policy at the local or federal levels. The order and content of each type of information on the five policy aspects were randomised. | Economic costs: No information vs. Consumer bill savings vs. Consumer bill increase of less than $100 per year vs. Consumer bill increase of more than $100 per year;  Consequences for the gas and renewable energy industries: No information vs. Natural gas industry opposition vs. Renewable energy industry support;  Health effects of natural gas use: No information vs. Reduce indoor air pollution that can damage individuals vs. Reduce indoor air pollution that can damage children;  Climate change implications of natural gas use: No information vs. Reduce greenhouse gas emissions vs. Burning natural gas helps to cut greenhouse gas vs. Both;  Political support for the policy: Local level vs. Federal level | Support for natural gas ban |
| Meeks, 2023 | To explore how partisans' trust in the political candidate interact with the frames and affect levels of climate-change political participation, behaviours, salience, hope, and efficacy. | United States | Quantitative randomised controlled trial | N = 342; Female: 50·0%; Mean age = 46·2 | Participants were randomly assigned to a control condition where no information is presented, or to the message conditions where they were exposed to a candidate's campaign speech (320-350 words) about climate change and mitigation strategies emphasising either a health or a national security frame. | No-message control vs. Health frame vs. National security frame | Political participation regarding climate change; Green behavioural intention; Issue salience; Hope; Efficacy |
| Myers et al., 2012 | To investigate how unique audience segments emotionally react to news articles crafted to reflect three distinct climate change message frames: (1) a traditional environmental frame, (2) a national security frame, and (3) a health frame. | United States | Quantitative randomised controlled trial | N = 1127 | Participants were randomly assigned to read one of three articles each of around 610 words, emphasising different dimensions of climate change and mitigation strategies (environment, national security, health). | Environment frame (emphasised the consequences of climate change to ecosystems, and the benefits to ecosystems of adaptation and mitigation-related actions) vs. National security frame (highlighted the risks of climate change to national security, and the benefits to national security of adaptation and mitigation-related actions) vs. Health frame (stressed the health risks associated with climate change, and the potential benefits to health of adaptation and mitigation-related actions) | Selection of sentences that made participants feel hopeful or angry |
| Myrick, 2019 | To test the different effects of environmental and health message frames, message source, and use of visuals, while also comparing different theoretical explanations for frame and message feature effects on audience attitudes toward climate change mitigation policy. | United States | Quantitative randomised controlled trial | Adults; N = 400; Female: 47·0%; Mean age = 54·3 | All participants were presented three mock Twitter pages about the effects of climate change related to heat, air quality, and water quality. Participants were randomly assigned to read the Twitter pages designed to look as if they either came from the Obama White House’s official Twitter page (liberal source) or the official Twitter page of Clear Path (conservative source). Participants were also randomly assigned to read either the environmental frame or the health frame. | Source: Liberal vs. Conservative;  Frame: Environmental frame (focused on negative consequences related to nature) vs. Health frame (emphasized negative outcomes of climate change related to health problems) | Policy support attitudes |
| Nabi et al., 2018 | To investigate the role of emotion, fear, and hope, in the gain/loss framing of environmental policy initiatives. | United States | Quantitative randomised controlled trial | University students; N = 337; Female: 77·0%; Mean age = 20·0 | Participants read two messages presented as very recent Los Angeles Times new stories. The first message (600 words) was either a threatening or a nonthreatening message about climate change. The threat-based message described the health threats of climate change in the United States. The control message offered a brief biography of the report’s lead editor and a chronology of federal climate change reporting over the past 20 years. The second message (500 words) was a solutions message framed in terms of either gain or loss. This message described two policy initiatives under consideration in California that could help stop climate change and benefit public health: (a) redesigning cities and towns to make them more walkable, bikeable, and conducive to taking public transit and (b) increasing the speed of the current transition to cleaner sources of energy. | First message about climate change: Threat (highlighted the ways in which climate change threatens national health) vs. Control (history of federal climate change reports);  Second message about mitigation strategies and health effects: Gain frame vs. Loss frame | Message assessment; Fear; Hope; Attitudes toward climate change policies; Advocacy behaviour |
| Orset, 2019 | To evaluate the willingness to pay for four means of transport: two high-emission vehicles (diesel taxi and diesel personal vehicle) and two low-emission vehicles (rented electric vehicle and public transport). | France | Quantitative non-randomised study | Adults; N = 342; Female: 51·7%; Age range: 20-80 | All participants read seven successive messages (around 300 words in total) emphasising health and environmental impacts of air pollution. One group first received information on the negative impact of air pollution on health and then on the environment, while another group first received information on the negative impact on the environment and then on health. | Seven messages | Willingness to pay for each means of transport |
| Petrovic et al., 2014 (Study 1) | To examine whether framing fossil fuel emissions in terms of public health versus climate change differentially influenced attitudes towards mitigation efforts. | United States | Quantitative randomised controlled trial | N = 402; Female: 58·0%; Mean age = 36·0 | Participants were randomised to read one of two versions of a statement (55-70 words) framing fossil fuel burning as a public health or climate change threat. | Climate frame vs. Health frame | Concern; Support for mitigation efforts |
| Petrovic et al., 2014 (Study 2) | To examine whether framing fossil fuel emissions in terms of public health versus climate change differentially influenced attitudes towards mitigation efforts. | United States | Quantitative randomised controlled trial | N = 397; Female: 33·0%; Mean age = 31·0 | Participants were randomised to read one of two versions of a statement (10-30 words) about harm to human health resulting from air pollution, with one specifying the pollution to originate from “fossil fuels (coal, oil, and natural gas)”. | Fossil fuel frame vs. Air pollution frame | Degree to which participants agreed with the message |
| Petrovic et al., 2014 (Study 3) | To examine whether framing fossil fuel emissions in terms of public health versus climate change differentially influenced attitudes towards mitigation efforts. | United States | Quantitative randomised controlled trial | N = 794; Female: 40·0%; Mean age = 34·0 | Participants were randomised to read one of two versions of a statement (15-25 words) framing air pollution as a public health or climate change threat, omitting references to fossil fuels. | Climate frame vs. Health frame | Degree to which participants agree with the message; Support for mitigation efforts; Personal responsibility; Willingness to donate to a charity; Willingness to pay more for electricity |
| Poortinga et al., 2023 | To explore factors and framing effects in public support for climate change mitigation policies in the UK. | United Kingdom | Quantitative randomised controlled trial | N = 5665; Female: 51·3%; Age range: 16+ | Participants were presented descriptions of four out of eight net zero policies, and were randomly presented one of four frames of the policies: (1) neutral frame, (2) climate change frame, (3) health frame, or (4) economic frame. | Neutral frame (contained a technical description of the policies) vs. Climate change frame (presented potential climate impact of the policy) vs. Health frame (presented potential health, safety, or general lifestyle impacts of the policy) vs. Economic frame (presented potential financial impacts of the policy) | Support for net zero policies |
| Rosen et al., 2021 | To examine the effect and efficacy of visual designs for messages about poor air quality. | United States | Quantitative randomised controlled trial | College students; N = 95; Female: 60·0%; Age range: 18-34; Mean age = 21·0 | Participants read a message on air quality and the health impacts of exposure to polluted air. Participants were randomly assigned to read either a high-efficacy or a low-efficacy message. | High-efficacy frame (described health impacts of air pollution and easy precautions one could take to decreased the risk of adverse health outcomes resulting from wildfire smoke exposure) vs. Low-efficacy frame (described health impacts of air pollution) | Protective behavioural intent; Source credibility |
| Schuldt et al., 2021 | To investigate the possible interactive effects of two types of climate framing devices on public's responses to environmental risks. | United States | Quantitative randomised controlled trial | N = 602; Female: 43·5%; Mean age = 34·0 | Participants read a short news article (about 200 words) that described the linkages between carbon emissions, marine ecosystem changes, and disease-causing pathogens afflicting oyster populations in the US Pacific Northwest. Depending on the condition, the article emphasised either the consequences for oyster health, or in addition the consequences for human health (via consumption of raw or undercooked oysters). Also varied was whether Vibrio outbreaks were said to be due to global warming or climate change. In addition, a control group was not exposed to any news article. | Oyster health frame (emphasised the consequences for oyster health) vs. Public health frame (emphasised the consequences for human health via consumption of raw or undercooked oysters) | Support for climate change policy; Support for marine disease policy; Causal attribution; Belief in global climate change |
| Shapiro & Bolsen, 2019 (Study 2) | To assess the impact of frames that accentuate the costs and benefits of the nation's coal usage on individuals' support for coal-based energy development. | South Korea | Quantitative randomised controlled trial | N = 500 | Participants were randomly assigned to receive no message, or to read a fabricated news article (60-100 words) about coal use, emphasising either its health costs, economical benefits, or both. | Control (no message) vs. Coal costs frame (health harm of coal use) vs. Coal benefits frame (economical benefits of coal use) vs. Competitive frame (combined coal costs and coal benefits) | Support for the increased development of coal; Perceived impact of increased coal use on the price of energy; Perceived impact of increased coal use on health problems |
| Sol Hart & Feldman, 2018 | To examine how discussing emissions in terms of climate change or air pollution, and in terms of health or environmental impacts, influenced individuals with different partisan affiliations. | United States | Quantitative randomised controlled trial | Adults; N = 1000; Female: 53·0%; Mean age = 47·3 | All participants read a statement (15 words) about power plant emission effects, emphasising general impacts (air pollution or climate change) and specific impacts (human health or environment). | General impact: Air pollution frame vs. Climate change frame;  Specific impact: Human health frame vs. Environment frame | Belief in negative impacts of power plants; Support for government action |
| Sol Hart & Feldman, 2021 | To examine how framing power plant emissions in terms of air pollution or climate change, and in terms of health or environmental impacts, influences perceived benefits and costs of policies to reduce emissions and intentions to take political action that supports such policies. | United States | Quantitative randomised controlled trial | Adults; N = 1000; Female: 54·0%; Mean age = 46·8 | All participants read a statement (15 words) about power plant emission effects, emphasising general impacts (air pollution or climate change) and specific impacts (human health or environment). | General impact: Air pollution frame vs. Climate change frame;  Specific impact: Human health frame vs. Environment frame | Perceived benefit of government action; Perceived cost of government action; Intended political action |
| Stevenson et al., 2018 | To examine how message framing may affect emotional responses to climate change and subsequent individual and collective action among adolescents. | United States | Quantitative randomised controlled trial | High school agriculture students; N = 950; Female: 42·6% | Students were randomly presented with a climate news story (about 600 words) presented in one of four frames: agriculture, community, health, and environment. Each article began with a quote to introduce the idea that climate change would have significant impacts on the environment, agricultural systems, communities, and public health, respectively. The next section gave more detail on the expected impacts. The final section outlined co-benefits between reducing emissions and the topic of the frame. | Agriculture frame vs. Community frame vs. Health frame vs. Environment frame | Worry; Hope; Support for adaptation measures; Support for mitigation measures; Intentions to participate in individual actions |
| Stokes & Warshaw, 2017 | To examine whether the design and framing of renewable portfolio standard policies affect public opinion. | United States | Quantitative randomised controlled trial | Adults; N = 2500; Female: 54%; Age: 53% under 44 | All participants read a message about a hypothetical renewable energy bill. Those in the treatment condition then read a statement about how the bill would reduce harmful air pollution in their state, including toxins such as mercury. Those in the control condition did not receive any statement about air pollution or health. | Health (described how the bill will reduce harmful air pollution including toxins such as mercury) vs. Control (no information about air pollution or health) | Support for the hypothetical renewable energy bill |
| Tang et al., 2024 | To examine the effects of vicarious message interactivity in promoting actions against climate change and the underlying mechanisms behind this effect. | Mainland China | Quantitative randomised controlled trial | Adults; N = 236; Female: 64·8%; Age range: 19-57; Mean age = 29·7 | Participants read a mock Weibo post about climate change and its health impacts. Participants were randomised to two conditions: (1) the static condition where the post plainly described the threats posed by climate change and measures that can help address it, or (2) a vicarious interactive condition which presented a conversation between two Weibo users discussing the same topic. | Static message (a post created by an individual plainly describing the threats posed by climate change and measures that can help address it) vs. Interactive message (a conversation between two Weibo users discussing the same topic) | Psychological reactance; Message elaboration; Intention to take actions against climate change |
| Thompson et al., 2011 | To explore the interactive effects of dispositional threat orientation, type of message, and having children on reactions to a message about exposure to bisphenol A in plastics. | United States | Quantitative randomised controlled trial | Adults; N = 200; Female: 46·0%; Age range: 20-85; Mean age = 46·5 | Participants were assigned to read one of two messages regarding the dangers of Bisphenol A: plain message or fear arousal message. The plain message described risks associated with Bisphenol A and preventive measures. The fear arousal message contained additional information emphasising that the chemical is a health danger to ﻿oneself and one’s family. Cancer, diabetes, and other serious health problems were mentioned. | Plain message (described basic information on Bisphenol A risks and preventive measures) vs. Fear arousal message (included additional information emphasising Bisphenol A as a health danger to oneself and one's family, contributing to serious health problems including cancer and diabetes) | Reactions to message (combined score on perceived susceptibility to Bisphenol A effects, negative emotions, behavioural intentions to engage in protection) |
| Walker et al., 2018 (Study 1) | To explore whether and when non-climate frames can lead to greater support for climate policy relative to climate frames. | United Kingdom | Quantitative randomised controlled trial | University students; N = 240; Female: 64·6% | Participants were randomly assigned to one of four framing conditions, which presented identical information about car use reduction policy using either a public health frame, a climate change frame, a climate change followed by public health frame, or a public health followed by climate change frame. | Public health frame vs. Climate change frame vs. Climate change followed by public health frame vs. Public health followed by climate change frame | Support for car use reduction policy |
| Walker et al., 2018 (Study 2) | To explore whether and when non-climate frames can lead to greater support for climate policy relative to climate frames. | United Kingdom | Quantitative randomised controlled trial | University students; N = 59; Female: 76·3% | Participants were randomly allocated to two framing conditions, emphasising the benefits of two policies (reducing the national speed limit and increasing the price of fuel) for either public health or climate change. | Public health frame vs. Climate change frame | Support for the two policies |
| Wu et al., 2021 | To test the effects of air quality information on individuals' risk perception and precaution intention. | Mainland China | Quantitative randomised controlled trial | Adults; N = 150; Female: 45·3%; Age range: 18-57; Mean age = 29·7 | Participants were randomly assigned to view a message (20-50 words) about air quality and its impact on health with either a neutral or a negative descriptor, and warning either a vague or a specific target group. | Descriptor valence: Neutral (moderately polluted) vs. Negative (unhealthy);  Target group: Vague (sensitive individuals) vs. Specific (children, seniors, pregnant women, and people with respiratory diseases) | Self-risk perception of smog; Risk perception for others; Third-person perception (risk perception for others minus self-risk perception); Precaution intention |
| Wynes et al., 2021 | To analyse the effectiveness of communicating using different frames on pro-climate tweeting. | Canada | Mixed methods study | Members of Canadian Parliament; N = 335; Female: 27·2% | Members of Canadian Parliament were asked by constituents to post a pro-climate message to their Twitter account. The suggested tweets (20-30 words) represented either a public health frame or an environmental frame. | Public health frame vs. Environmental frame | Frequency of pro-climate tweets |

1. Bernauer T, McGrath LF. Simple reframing unlikely to boost public support for climate policy. Nat Clim Chang. 2016;6(7):680–3.

2. Buchanan T, Ackland J, Lloyd S, van der Linden S, De-Wit L. Clear consensus among international public for government action at COP26: patriotic and public health frames produce marginal gains in support [Internet]. Vol. 170, Climatic Change. Springer Netherlands; 2022. 24 p. Available from: https://doi.org/10.1007/s10584-021-03262-2

3. Cameron L, Rocque R, Penner K, Mauro I. Evidence-based communication on climate change and health: Testing videos, text, and maps on climate change and Lyme disease in Manitoba, Canada. PLoS One. 2021;16(6):e0252952.

4. Campbell E. Health Professionals as Advocates for Climate and Health Solutions: The Influence of Social Norms on Health Professional Engagement in and Public Support for Climate Advocacy. George Mason University; 2023.

5. Connor P, Harris E, Guy S, Fernando J, Shank DB, Kurz T, et al. Interpersonal communication about climate change: how messages change when communicated through simulated online social networks. Clim Change. 2016;136:463–76.

6. Dasandi N, Graham H, Hudson D, Jankin S, VanHeerde-Hudson J, Watts N. Positive, global, and health or environment framing bolsters public support for climate policies. Commun Earth Environ. 2022;3:239.

7. Delmas MA, Kohli A. Engagement With Air Quality Information: Stated Versus Revealed Preferences. Organ Environ. 2021;34(3):413–34.

8. Feldman L, Sol Hart P. Climate change as a polarizing cue: Framing effects on public support for low-carbon energy policies. Glob Environ Chang [Internet]. 2018;51:54–66. Available from: https://doi.org/10.1016/j.gloenvcha.2018.05.004

9. Feldman L, Sol Hart P. Broadening exposure to climate change news? How framing and political orientation interact to influence selective exposure. J Commun. 2018;68:503–24.

10. Godawska J. Framing effect and public support for the environmental policy. Econ Environ. 2020;1(72):24–39.

11. Hubbert B, Ahmed M, Kotcher J, Maibach E, Sarfaty M. Recruiting health professionals as sustainability advocates. Lancet Planet Heal [Internet]. 2020;4(10):e445–6. Available from: http://dx.doi.org/10.1016/S2542-5196(20)30225-4

12. Jasemzadeh M, Khafaie MA, Jaafarzadeh N, Araban M. Effectiveness of a theory-based mobile phone text message intervention for improving protective behaviors of pregnant women against air pollution: a randomized controlled trial. Environ Sci Pollut Res. 2018;25:6648–55.

13. Joshi A. Motivating sustainable behaviors by framing biodiversity loss as a public health risk. J Risk Res [Internet]. 2022;25(2):156–75. Available from: https://doi.org/10.1080/13669877.2021.1913634

14. Kim SC, Pei D, Kotcher JE, Myers TA. Predicting Responses to Climate Change Health Impact Messages From Political Ideology and Health Status: Cognitive Appraisals and Emotional Reactions as Mediators. Environ Behav. 2021;53(10):1095–117.

15. Kotcher J, Feldman L, Luong KT, Wyatt J, Maibach E. Advocacy messages about climate and health are more effective when they include information about risks, solutions, and a normative appeal: Evidence from a conjoint experiment. J Clim Chang Heal [Internet]. 2021;3:100030. Available from: https://doi.org/10.1016/j.joclim.2021.100030

16. Kotcher J, Luong K, Charles J, Gould R, Maibach E. Calling attention to opponents of climate action in climate and health messaging. Lancet Planet Heal [Internet]. 2023;7:e938–46. Available from: http://dx.doi.org/10.1016/S2542-5196(23)00217-6

17. Kotcher J, Maibach E, Choi WT. Fossil fuels are harming our brains: Identifying key messages about the health effects of air pollution from fossil fuels. BMC Public Health. 2019;19:1079.

18. Kotcher J, Maibach E, Montoro M, Hassol SJ. How Americans Respond to Information About Global Warming’s Health Impacts: Evidence From a National Survey Experiment. GeoHealth. 2018;2:262–75.

19. Landrum AR, Stecuła DA, Motta M. Combating climate-induced health threats through Co-Constitutive Risk (CCR) Messaging: A One Health Communication approach. PLoS Negl Trop Dis [Internet]. 2024;18(12):e0012676. Available from: http://dx.doi.org/10.1371/journal.pntd.0012676

20. Levine AS, Kline R. A new approach for evaluating climate change communication. Clim Change. 2017;142:301–9.

21. Li SCS, Zeng HK, Lo SY. Young Adults’ Intentions toward the Prevention of Microplastic Pollution in Taiwan: Examining Personality and Information Processing in Fear-Appeal Communication. Sustainability. 2022;14:14336.

22. Liu W, Shao W, Wang Q. Does fear of the new coronavirus lead to low-carbon behaviors: The moderating effect of outcome framing. Risk Manag Healthc Policy. 2021;14:4185–97.

23. Liu Z, Yang JZ. Communicating Per- and Polyfluoroalkyl Substances (PFAS) Contamination to the Public Through Personal Relevance. J Health Commun [Internet]. 2023;28:73–81. Available from: https://doi.org/10.1080/10810730.2023.2183284

24. MacInnis B, Krosnick JA, Abeles A, Caldwell MR, Prahler E, Dunne DD. The American public’s preference for preparation for the possible effects of global warming: impact of communication strategies. Clim Change. 2015;128:17–33.

25. Maibach EW, Nisbet M, Baldwin P, Akerlof K, Diao G. Reframing climate change as a public health issue: an exploratory study of public reactions. BMC Public Health [Internet]. 2010;10:299. Available from: http://www.biomedcentral.com/1471-2458/10/299

26. McComas KA, Schuldt JP, Burge CA, Roh S. Communicating about marine disease: The effects of message frames on policy support. Mar Policy [Internet]. 2015;57:45–52. Available from: http://dx.doi.org/10.1016/j.marpol.2015.02.012

27. McLean E V., Whang T, Yang J. Clearing the air: Public health concerns and support for natural gas restrictions in the United States. Energy Res Soc Sci [Internet]. 2024;108:103404. Available from: https://doi.org/10.1016/j.erss.2023.103404

28. Meeks L. Red, Blue, and Green: Examining the Effects of Framing and Source Trust on Partisans’ Climate-Change Beliefs. In: Political Communication, Culture, and Society. Taylor & Francis; 2023. p. 150–66.

29. Myers TA, Nisbet MC, Maibach EW, Leiserowitz AA. A public health frame arouses hopeful emotions about climate change: A Letter. Clim Change. 2012;113:1105–12.

30. Myrick JG. Comparing Theoretical Explanations for the Empirical Effects of Presenting Climate Change as a Health Issue on Social Media. In: Climate Change, Media and Culture: Critical Issues in Global Environmental Communication. Emerald Group Publishing Limited; 2019. p. 33–52.

31. Nabi RL, Gustafson A, Jensen R. Framing Climate Change: Exploring the Role of Emotion in Generating Advocacy Behavior. Sci Commun. 2018;40(4):442–68.

32. Orset C. How Do Travellers Respond to Health and Environmental Policies to Reduce Air Pollution? Ecol Econ [Internet]. 2019;156:68–82. Available from: https://doi.org/10.1016/j.ecolecon.2018.08.016

33. Petrovic N, Madrigano J, Zaval L. Motivating mitigation: when health matters more than climate change. Clim Change. 2014;126:245–54.

34. Poortinga W, Whitmarsh L, Steentjes K, Gray E, Thompson S, Brisley R. Factors and framing effects in support for net zero policies in the United Kingdom. Front Psychol. 2023;14:1287188.

35. Rosen Z, Bice C, Scott S. Visualizing the invisible: Visual-based design and efficacy in air quality messaging. Int J Environ Res Public Health. 2021;18:10882.

36. Schuldt JP, McComas KA, Burge CA. Intersecting frames in communicating environmental risk and uncertainty. J Risk Res [Internet]. 2021;24(5):562–73. Available from: http://doi.org/10.1080/13669877.2017.1382559

37. Shapiro MA, Bolsen T. Korean perceptions of transboundary air pollution and domestic coal development: Two framing experiments. Energy Policy [Internet]. 2019;126:333–42. Available from: https://doi.org/10.1016/j.enpol.2018.11.013

38. Sol Hart P, Feldman L. Would it be better to not talk about climate change? The impact of climate change and air pollution frames on support for regulating power plant emissions. J Environ Psychol [Internet]. 2018;60:1–8. Available from: https://doi.org/10.1016/j.jenvp.2018.08.013

39. Sol Hart P, Feldman L. The Benefit of Focusing on Air Pollution Instead of Climate Change: How Discussing Power Plant Emissions in the Context of Air Pollution, Rather than Climate Change, Influences Perceived Benefits, Costs, and Political Action for Policies to Limit Emission. Sci Commun. 2021;43(2):199–224.

40. Stevenson KT, King TL, Selm KR, Peterson MN, Monroe MC. Framing climate change communication to prompt individual and collective action among adolescents from agricultural communities. Environ Educ Res [Internet]. 2018;24(3):365–77. Available from: http://dx.doi.org/10.1080/13504622.2017.1318114

41. Stokes LC, Warshaw C. Renewable energy policy design and framing influence public support in the United States. Nat Energy. 2017;2:17107.

42. Tang H, Chen L, Liu S, Tan X, Li Y. Reconsidering the Effectiveness of Fear Appeals: An Experimental Study of Interactive Fear Messaging to Promote Positive Actions on Climate Change. J Health Commun [Internet]. 2024;29:57–67. Available from: https://doi.org/10.1080/10810730.2024.2360025

43. Thompson SC, Schlehofer MM, Gonzalez A, Denison E. Reactions to a health threat: Dispositional threat orientations and message characteristics. Br J Health Psychol. 2011;16:344–58.

44. Walker BJA, Kurz T, Russel D. Towards an understanding of when non-climate frames can generate public support for climate change policy. Environ Behav. 2018;50(7):781–806.

45. Wu Y, Zhang L, Wang J, Mou Y. Communicating air quality index information: Effects of different styles on individuals’ risk perception and precaution intention. Int J Environ Res Public Health. 2021;18:10542.

46. Wynes S, Kotcher J, Donner SD. Can citizen pressure influence politicians’ communication about climate change? Results from a field experiment. Clim Change. 2021;168:6.

***Electronic Supplementary Material 3*: Quality assessment**

|  | | **Methodological quality criteria** | | | | |
| --- | --- | --- | --- | --- | --- | --- |
| **Quantitative randomised controlled trials** | | **Is randomisation appropriately performed?** | **Are the groups comparable at baseline?** | **Are there complete outcome data?** | **Are outcome assessors blinded to the intervention provided?** | **Did the participants adhere to the assigned intervention?** |
|  | Bernauer & McGrath, 2016 (Study 2) | Can't tell | Yes | Yes | Can't tell | Can't tell |
|  | Buchanan et al., 2022 | Can't tell | Can't tell | Yes | Can't tell | Can't tell |
|  | Campbell, 2023 (Study 1) | Can't tell | Yes | Yes | Can't tell | Can't tell |
|  | Campbell, 2023 (Study 2) | Can't tell | Yes | Can't tell | Can't tell | Can't tell |
|  | Connor et al., 2016 | Can't tell | Can't tell | Yes | Yes | Yes |
|  | Delmas & Kohli, 2021 (Study 1) | Can't tell | Can't tell | Yes | Can't tell | Yes |
|  | Delmas & Kohli, 2021 (Study 2) | Can't tell | Can't tell | Yes | Can't tell | Can't tell |
|  | Feldman & Sol Hart, 2018a (Study 2) | Can't tell | Can't tell | Yes | Can't tell | Yes |
|  | Feldman & Sol Hart, 2018b | Can't tell | Can't tell | Yes | Can't tell | Yes |
|  | Godawska, 2020 | Can't tell | Can't tell | Can't tell | Can't tell | Can't tell |
|  | Jasemzadeh et al., 2018 | Yes | Yes | Yes | Can't tell | Yes |
|  | Joshi, 2022 | Can't tell | Can't tell | No | Can't tell | Yes |
|  | Kotcher et al., 2018 | Can't tell | Can't tell | No | Can't tell | Yes |
|  | Kotcher et. al, 2023 | Can't tell | Can't tell | Yes | Can't tell | Yes |
|  | Landrum et al., 2024 | Can't tell | Yes | Yes | Can't tell | Can't tell |
|  | Levine & Kline, 2017 (Study 2) | Can't tell | Can't tell | Yes | Can't tell | Can't tell |
|  | Levine & Kline, 2017 (Study 3) | Can't tell | Yes | Yes | Can't tell | Can't tell |
|  | Levine & Kline, 2017 (Study 4) | Can't tell | Yes | Yes | Can't tell | Can't tell |
|  | Li et al., 2022 | Can't tell | Can't tell | Yes | Can't tell | Yes |
|  | Liu et. al, 2021 (Study 2) | Can't tell | Can't tell | Yes | Can't tell | Yes |
|  | Liu & Yang, 2023 | Can't tell | Yes | Yes | Can't tell | Yes |
|  | MacInnis et al., 2015 (Study 2) | Can't tell | Can't tell | Yes | No | Can't tell |
|  | McComas et al., 2015 | Can't tell | Yes | Yes | Can't tell | Yes |
|  | McLean et al., 2024 | Can't tell | Can't tell | Yes | Can't tell | Yes |
|  | Meeks, 2023 | Can't tell | Can't tell | Yes | Can't tell | Yes |
|  | Myers et al., 2012 | Can't tell | Can't tell | Yes | Can't tell | Can't tell |
|  | Myrick, 2019 | Can't tell | Can't tell | Yes | Can't tell | Yes |
|  | Nabi et al., 2018 | Can't tell | Can't tell | Yes | Can't tell | Yes |
|  | Petrovic et al., 2014 (Study 1) | Can't tell | Can't tell | Yes | Can't tell | Yes |
|  | Petrovic et al., 2014 (Study 2) | Can't tell | Can't tell | Yes | Can't tell | Can't tell |
|  | Petrovic et al., 2014 (Study 3) | Can't tell | Can't tell | Yes | Can't tell | Can't tell |
|  | Poortinga et al., 2023 | Can't tell | Can't tell | Yes | Can't tell | Can't tell |
|  | Rosen et al., 2021 | Can't tell | Can't tell | Yes | Can't tell | Yes |
|  | Schuldt et al., 2021 | Can't tell | Can't tell | Yes | Can't tell | Can't tell |
|  | Shapiro & Bolsen, 2019 (Study 2) | Can't tell | Can't tell | Yes | Can't tell | Can't tell |
|  | Sol Hart & Feldman, 2018 | Can't tell | Yes | Yes | Can't tell | Yes |
|  | Sol Hart & Feldman, 2021 | Can't tell | Yes | Yes | Can't tell | Yes |
|  | Stevenson et al., 2018 | Can't tell | Can't tell | Yes | Can't tell | Can't tell |
|  | Stokes & Warshaw, 2017 | Can't tell | Can't tell | Yes | Can't tell | Can't tell |
|  | Tang et al., 2024 | Can't tell | Yes | Yes | Can't tell | Yes |
|  | Thompson et al., 2011 | Can't tell | Can't tell | Yes | Can't tell | Yes |
|  | Walker et al., 2018 (Study 1) | Can't tell | Can't tell | Yes | No | Can't tell |
|  | Walker et al., 2018 (Study 2) | Can't tell | Can't tell | Yes | Can't tell | Yes |
|  | Wu et al., 2021 | Can't tell | Yes | Yes | Can't tell | Yes |
| **Quantitative non-randomised studies** | | **Are the participants representative of the target population?** | **Are measurements appropriate regarding both the outcome and intervention (or exposure)?** | **Are there complete outcome data?** | **Are the confounders accounted for in the design and analysis?** | **During the study period, is the intervention administered (or exposure occurred) as intended?** |
|  | Dasandi et al., 2022 | Yes | Yes | Yes | Yes | Yes |
|  | Feldman & Sol Hart, 2018a (Study 1) | Yes | Yes | Yes | Yes | Yes |
|  | Kim et al., 2021 | Yes | Yes | Yes | Yes | Yes |
|  | Kotcher et al., 2019 | Yes | Yes | Yes | Yes | Yes |
|  | Kotcher et. al, 2021 | Yes | Yes | Yes | Yes | Yes |
|  | Orset, 2019 | Yes | Yes | Yes | Yes | Yes |
| **Mixed methods studies** | | **Is there an adequate rationale for using a mixed methods design to address the research question?** | **Are the different components of the study effectively integrated to answer the research question?** | **Are the outputs of the integration of qualitative and quantitative components adequately interpreted?** | **Are divergences and inconsistencies between quantitative and qualitative results adequately addressed?** | **Do the different components of the study adhere to the quality criteria of each tradition of the methods involved?** |
|  | Maibach et al., 2010 | Yes | Yes | Yes | Yes | Yes |
|  | Wynes et al., 2021 | Yes | Yes | Yes | Yes | Yes |
| **Qualitative studies** | | **Is the qualitative approach appropriate to answer the research question?** | **Are the qualitative data collection methods adequate to address the research question?** | **Are the findings adequately derived from the data?** | **Is the interpretation of results sufficiently substantiated by data?** | **Is there coherence between qualitative data sources, collection, analysis and interpretation?** |
|  | Cameron et al., 2021 | Yes | Yes | Yes | Yes | Yes |
|  | Hubbert et al., 2020 | Yes | Yes | Yes | Yes | Yes |
